# Extended low-resolution structure of a *Leptospira* antigen offers high bactericidal antibody accessibility amenable to vaccine design

**DOI:** 10.7554/eLife.30051

**Published:** 2017-12-06

**Authors:** Ching-Lin Hsieh, Christopher P Ptak, Andrew Tseng, Igor Massahiro de Souza Suguiura, Sean P McDonough, Tepyuda Sritrakul, Ting Li, Yi-Pin Lin, Richard E Gillilan, Robert E Oswald, Yung-Fu Chang

**Affiliations:** 1 Department of Population Medicine and Diagnostic Sciences, College of Veterinary MedicineCornell UniversityIthacaUnited States; 2 Department of Molecular Medicine, College of Veterinary MedicineCornell UniversityIthacaUnited States; 3 Department of Biomedical Sciences, College of Veterinary MedicineCornell UniversityIthacaUnited States; 4 Division of Infectious DiseaseWadsworth Center, New York State Department of HealthAlbanyUnited States; 5 Macromolecular Diffraction Facility at CHESS (MacCHESS)Cornell UniversityIthacaUnited States; J.W. Goethe-UniversityGermany

**Keywords:** LigB structure, mAb accessibility, vaccine design, Other

## Abstract

Pathogens rely on proteins embedded on their surface to perform tasks essential for host infection. These obligatory structures exposed to the host immune system provide important targets for rational vaccine design. Here, we use a systematically designed series of multi-domain constructs in combination with small angle X-ray scattering (SAXS) to determine the structure of the main immunoreactive region from a major antigen from *Leptospira interrogans*, LigB. An anti-LigB monoclonal antibody library exhibits cell binding and bactericidal activity with extensive domain coverage complementing the elongated architecture observed in the SAXS structure. Combining antigenic motifs in a single-domain chimeric immunoglobulin-like fold generated a vaccine that greatly enhances leptospiral protection over vaccination with single parent domains. Our study demonstrates how understanding an antigen’s structure and antibody accessible surfaces can guide the design and engineering of improved recombinant antigen-based vaccines.

## eLife digest

Vaccines encourage the immune system to develop a protection against disease-causing bacteria and viruses. Some types of immune cells release antibodies, which recognize particular proteins on the surface of the invading microbe. A vaccine that contains these surface proteins allows immune cells to develop the antibodies that can help to fight off an infection at a later date. Studying the shape and structure of the surface proteins can reveal how they are detected by our immune systems and can further be used to design more effective vaccines.

Leptospirosis is the most common bacterial disease to affect both humans and animals. The symptoms of the disease include fever, muscle pain and bleeding from the lungs. New vaccines against leptospirosis are desperately needed because current ones have severe side effects and do not fully protect against the disease. The most promising new vaccine candidates are the Lig proteins, which are found on the surface of leptospirosis causing bacteria cells, but little was known about their molecular structure.

The region of the Lig protein that is recognized by the immune system consists of a series of twelve connected ‘immunoglobulin-like’ domains. Hsieh, Ptak et al. used X-ray scattering to determine the structure of this region and found that the protein is highly elongated. Additional experiments showed that the individual domains provoke immune responses to different extents. Antibodies that can interact more strongly with the Lig protein were also better able to kill the bacteria. Based on this information, Hsieh, Ptak et al. combined parts of the individual domains that bind strongly to antibodies to design a new protein that, when used as a vaccine, protected hamsters against leptospirosis much better than other Lig protein-based vaccines.

Further engineering and testing are required to develop an optimized, commercial leptospirosis vaccine, but the work of Hsieh, Ptak et al. shows the effectiveness of structure-based vaccine design methods. In the future, similar methods could be used to develop better vaccines and treatments for other infectious diseases.

## Introduction

The molecular details of how surface antigens of a pathogen are exposed to the host’s defenses are highly relevant to rational vaccine development (Rappuoli et al., 2016; Dormitzer et al., 2012; Dormitzer et al., 2008). The structural context of immunologically accessible epitopes allows for the redesign of recombinant vaccine scaffolds to display the most antigenic surfaces (Correia et al., 2014; Malito et al., 2014). The exploration of recombinant strategies can be particularly fruitful when classical vaccine strategies provide weak protection. Currently available inactivated vaccines against leptospirosis, the most common bacterial zoonosis (Picardeau, 2017), are inadequate because of the severe side-effects and lack of cross-protection among pathogenic *Leptospira* species (Grassmann et al., 2017; Adler, 2015). Because the estimated worldwide burden of leptospirosis is over 1 million severe cases and ~60,000 deaths per year, advances in recombinant leptospiral vaccines are desperately needed and are likely to benefit from a structure-based antigen design strategy (Picardeau, 2017; Costa et al., 2015).

While several leptospiral antigens have been explored for use in vaccines, the most promising candidates have been derived from the *Leptospira* immunoglobulin-like (Lig) protein family (Ko et al., 2009; Grassmann et al., 2017; Cao et al., 2011; Yan et al., 2009; Chang et al., 2007). Lig proteins are present in only pathogenic species with LigB (but not LigA or LigC) being found in all pathogenic *Leptospira* genomes (McBride et al., 2009; Matsunaga et al., 2003), LigB’s expression during host invasion further suggests an important role in virulence (Lessa-Aquino et al., 2017; Choy et al., 2007). To promote infection, LigB can bind multiple blood factors and extracellular matrix molecules (ECMs) to facilitate immune system evasion and tissue colonization (Choy, 2012; Vieira et al., 2014; Figueira et al., 2011). The persistence and exposure of LigB on the leptospiral outer membrane during infection leaves an exploitable vulnerability. Recently, a hamster immunization study has suggested that LigB-derived vaccines have the potential to confer sterile immunity against leptospiral challenge (Conrad et al., 2017). The new study (Conrad et al., 2017) is inconsistent with earlier LigB vaccine studies which only confer partial protection (Yan et al., 2009; Silva et al., 2007) and raises the possibility of further improvements in LigB-derived vaccine efficiency through rational design.

Our understanding of the LigB structure is limited to the NMR structure of the individual Lig protein Ig-like domain (Ptak et al., 2014). LigB is attached to the leptospiral outer surface by a short N-terminal anchor, which is followed by a stretch of twelve consecutive Ig-like domains, and flanked at its C-terminus by an additional non-Ig-like domain (Figure 1—figure supplement 1A). Most host interactions with Lig protein have been identified within the Ig-like domain regions and previously reported LigB-based vaccines have targeted the Ig-like domain region (Yan et al., 2009; Conrad et al., 2017; Breda et al., 2015; Lin et al., 2009a). A more comprehensive understanding of the domain arrangement would provide a picture of the most accessible antigenic regions and a guide for structure-based vaccine design.

In this study, small angle X-ray scattering (SAXS) (Skou et al., 2014) was used to obtain a low-resolution solution structure of the Ig-like domain region’s architecture. The full LigB Ig-like domain region’s extended arrangement with notable bends encouraged the exploration of the highly-exposed surface for immunoreactivity with a library of anti-LigB monoclonal antibodies (mAbs). The capability of these mAbs to bind antigen and to adhere to the surface of pathogenic *Leptospira* was then correlated with their ability to kill these bacteria in the presence of serum complements. Finally, the identified mAb-reactive domains and previously obtained LigB12 (LigB 12th Ig-like domain) NMR structure informed the generation of chimeras on single Ig-like domain scaffolds capable of eliciting an immune response with either side of the β-sandwich domain. To illustrate the potential for rationally engineered antigens, a vaccine containing the chimera LigB10-B7-B7, which displays identified mAb-interacting surfaces from LigB7 and LigB10, offered greatly improved protection over LigB7 and LigB10 against leptospiral lethal challenge in hamsters. These findings provide a blueprint for combining immunoreactivity mapping from an mAb library and high-resolution structural information from NMR to engineer epitopes and improve the efficacy of LigB vaccines as well as recombinant vaccines from other pathogens.

## Results

### Extended structure of the LigB Ig-like domain region

To determine the architecture of the twelve Ig-like domain stretch in LigB, small angle X-ray scattering (SAXS) was used to generate low-resolution solution structures along a sliding multi-domain window. Because the 5-domain length contains four domain-domain linkers, the 5-domain structure proved to be optimal to define the relative angle of two neighboring domain-domain joints with one additional joint on each end (See Materials and methods for rationale; Figure 1—figure supplement 1).

**Figure 1.**
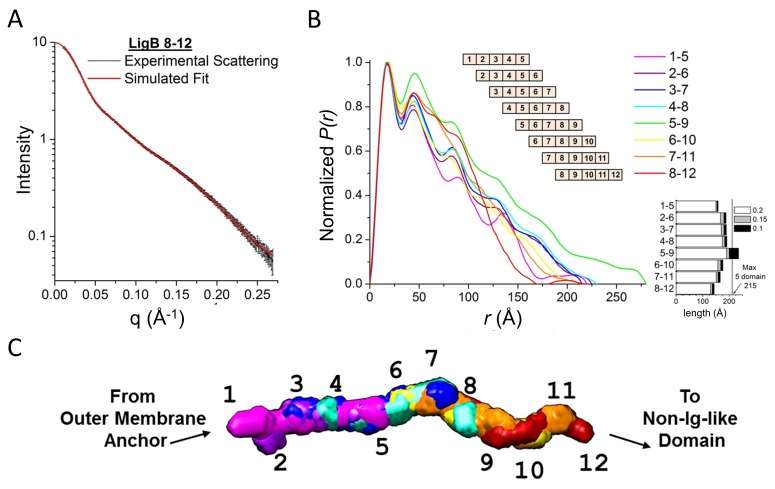
Full LigB Ig-like domain region (LigB1-12) determined from experimental SAXS data of 5-domain constructs. (**A**) The experimental scattering data for LigB8-12 (black) is shown with the simulated fit (red). The scattering curve is an average of 15 scans ± S.D. (**B**) The pair distance distributions, *P(r)*, were calculated from SAXS plots of all possible 5-domain LigB Ig-like domains using GNOM. A representation of the 5-domain constructs is shown in the inset. An estimate of construct length based on the longest atomic distances at 20%, 15%, and 10% max population height are shown. (**C**) DAMFILT envelopes were combined to create a representative envelope of the twelve Ig-like domains. Construct LigB5-9 was not included because the maximum distance distribution exceeds the expected length of a 5-domain construct. 10.7554/eLife.30051.006Figure 1—source data 1.LigB five domain construct SAXS profile values.

All eight possible 5-domain protein constructs (LigB1-5 to LigB8-12) were analyzed with SAXS (Figure 1; Figure 1—figure supplement 2). Guinier fits and Porod analysis suggested minimal aggregation for all but LigB5-9 (Figure 1—figure supplement 2B and Figure 1—source data 1). The simulated fits to the experimental SAXS curves for the eight 5-domain constructs (Figure 1A; Figure 1—figure supplement 2A) were used to generate the atomic distance distribution for the molecules (Figure 1B). The longest atomic pair distance deduced from the pair-distance distribution function (*P(r)*) is indicative of the length of the 5-domain proteins. The expected length of a fully extended arrangement of five folded LigB Ig-like domains is only slightly longer than the pair distance for most of the eight 5-domain proteins and their corresponding envelopes (Figure 1B; Figure 1—figure supplement 2C). LigB5-9 has an atomic pair distance that exceeds what is possible for a folded 5-domain monomer (in agreement with aggregation indicated by the difference between experimental and predicted molecular weights, Figure 1—source data 1) and was excluded from the final model.

**Figure 1—figure supplement 1.**
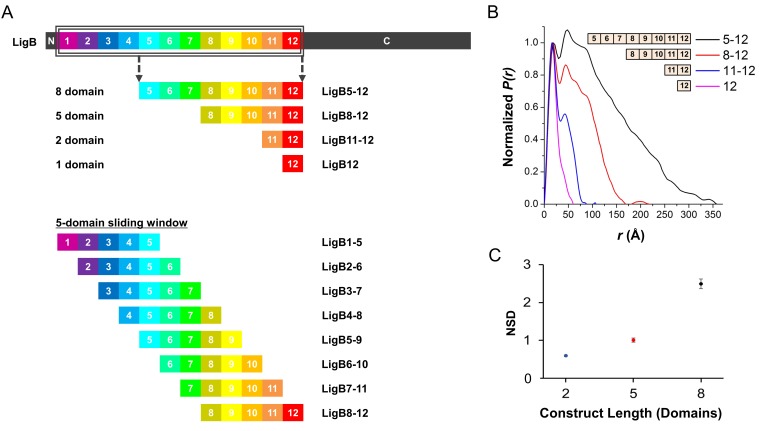
SAXS construct design. (**A**) Schematic of the full length LigB protein. The set of LigB constructs used to test the optimal number of Ig-like domains and the set of 5-domain constructs used for SAXS analysis. (**B**) SAXS atomic pair distance distributions, *P(r)*, for 8, 5, 2, and 1 domain constructs (LigB5-12, LigB8-12, LigB11-12, and LigB12, respectively) were calculated from SAXS plots using GNOM. The *P(r)* was normalized at the first peak (15–20 Å) for each of the distributions. (**C**) The normalized spatial discrepancy (NSD) derived using the DAMAVER program suite is plotted for multi-domain LigB constructs shown in B. Error bars show S.D. for the for the 10 computed bead models. NSD values > 1 are indicative of heterogeneity in the DAMAVER models.

**Figure 1—figure supplement 2.**
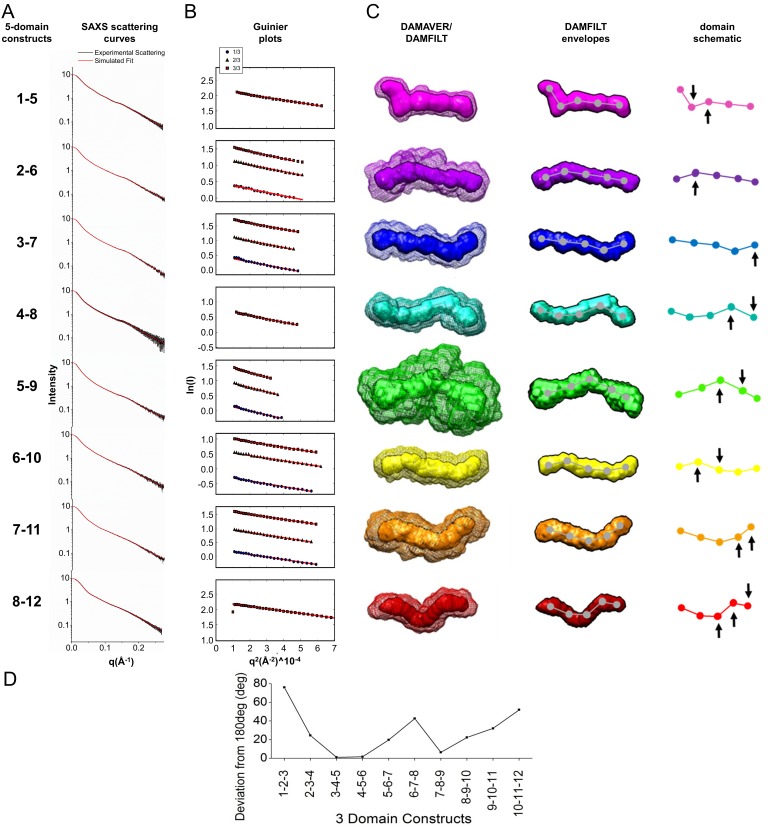
Experimental SAXS data and structures for all LigB Ig-like domain 5-domain constructs. (**A**) The experimental scattering data (black line, error bars) are shown for solutions containing LigB1-5, LigB2-6, LigB3-7, LigB4-8, LigB5-9, LigB6-10, LigB7-11, and LigB8-12. The scattering curves are an average of 15 scans ± S.D. A simulated curve (red line) was fit to the data. (**B**) Guinier plots for the experimental scattering data were calculated with the RAW software using standard cutoffs. Fits (red lines) indicate a short but reasonable linear region characteristic of elongated structures. For data collected on samples after dilution, the relative concentration is indicated as the fraction of original protein stock (3/3, 2/3, 1/3; exact concentrations listed in Figure 1—source data 1). (**C**) Curves fitted to the SAXS data were used to generate *ab initio* models for all possible 5-domain stretches of LigB Ig-like domains with DAMMIF. DAMAVER/DAMFILT envelopes, DAMFILT envelopes, and dots depicting domain positions are arranged in rows for the 5-domain constructs. Envelopes were positioned to draw attention to the most significant bends within the stretch of domains. Arrows indicate positions where bending is occurring on the dot representations. (**D**) The angles created from three neighboring dots within the dot representations were averaged and illustrate the degree to which the neighboring domains deviate from a linear (180°) arrangement.

Ambiguity assessment using the AMBIMETER program (Petoukhov and Svergun, 2015) shows that the first six structures (LigB1-5 to LigB6-10) have a high degree of uniqueness with ambiguity scores of 0.3 or lower. The last two structures (LigB7-11 and LigB8-12) have scores of 1.7 and 1.4 respectively, which indicates the potential for shape ambiguity. Each of the eight 5-domain SAXS envelopes exhibited distinct structures with a variety of domain-domain angles (Figure 1—figure supplement 2D). A significant degree of bending is present between the first three domains. The stretch of domains between LigB3-6 is particularly straight. Slight bending in the angle between domains in the final five domain stretch produces a gentle spiral shape. By aligning the bends in the four shared domains of neighboring 5-domain regions, a best fit structure was generated for the full twelve Ig-like domains of LigB (Figure 1C). Overall the LigB1-12 structure is only 16% shorter than a fully extended model structure of twelve folded domains. The SAXS-derived structure of LigB1-12 demonstrates that most of the individual Ig-like domains exhibit a high degree of exposed surface area and suggests a high degree of accessibility to host interactions. The extensive exposed surface area is a consequence of the unexpected rigid, rod-like structure, with several well-defined kinks.

### Creation of mAb libraries against LigB

**Figure 2.**
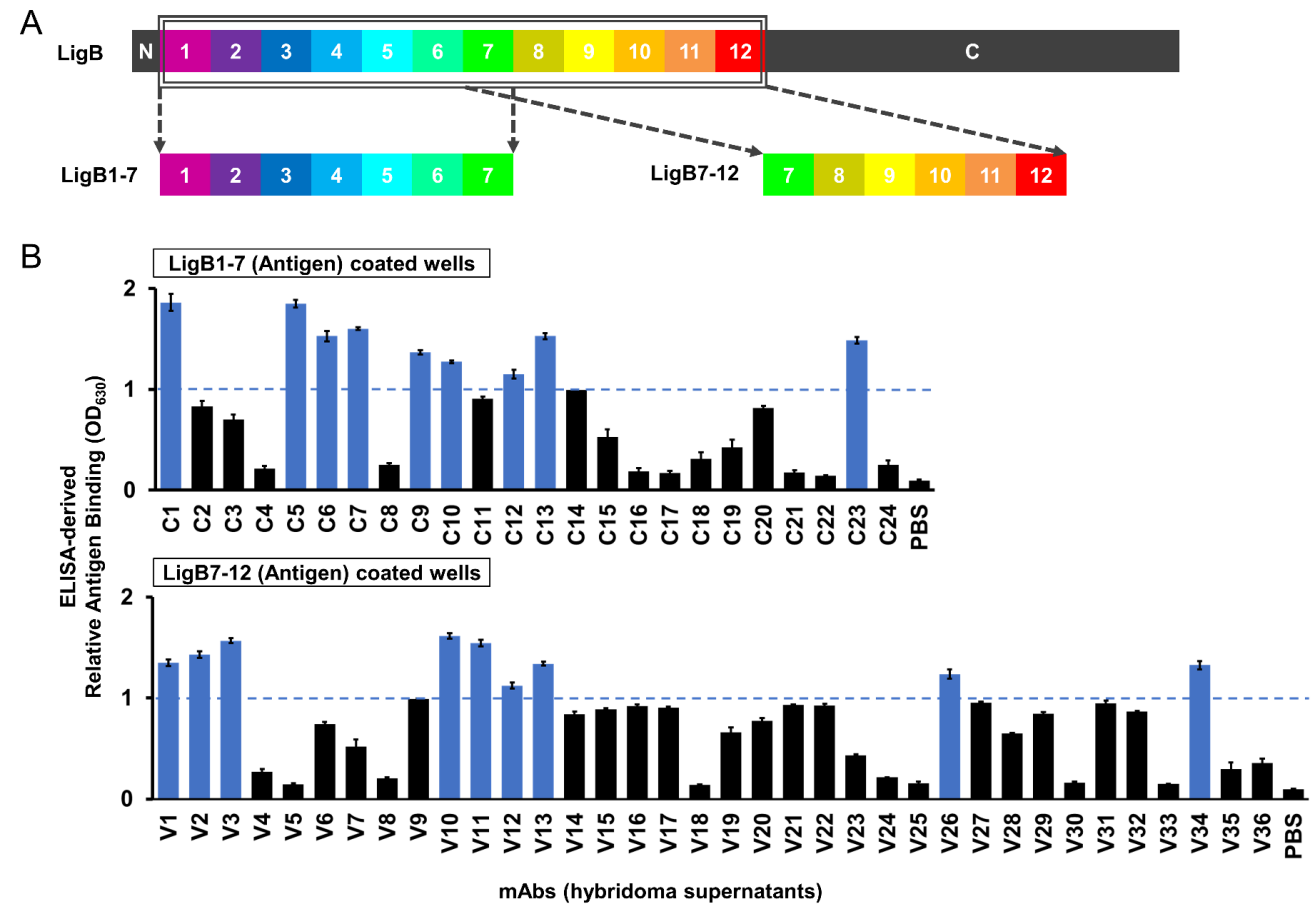
ELISA screen of anti-LigB mAb library. (**A**) Depiction of the full length LigB antigen and LigB-derived antigens used for mAb generation. (**B**) The degree of mAb binding to the respective LigB antigens was indirectly measured by colorimetic (λ_630_) TMB-ELISA using rabbit anti-mouse IgG antibody conjugated with HRP (1:5000). Each value represents the mean ±S.D. from three individual trials of two replicates. Additional mAb characterization was limited to mAbs with binding efficiencies resulting in OD_630_ >1.0 (blue).

To explore the exposure of the LigB Ig-like domain region to a host immune response, two purified LigB truncations, LigB1-7 and LigB7-12 (Figure 2A), were used to generate two sets of hybridoma cell lines for mAb production: library C (24 mAbs) and library V (36 mAbs), respectively. Using ELISA, hybridoma supernatants containing anti-LigB mAbs were qualitatively screened for the ability to bind to their respective LigB truncations (Figure 2B). Based on the distribution of antigen binding efficiencies, a threshold level was set to OD_630_ = 1.0 (Figure 2—figure supplement 1). Only mAbs with binding above the threshold level were purified for further characterization of binding properties and bactericidal activity. Screening of library V required an additional twelve mAbs in order to equal the nine threshold-level mAbs identified in the library C screen.

**Figure 2—figure supplement 1.**
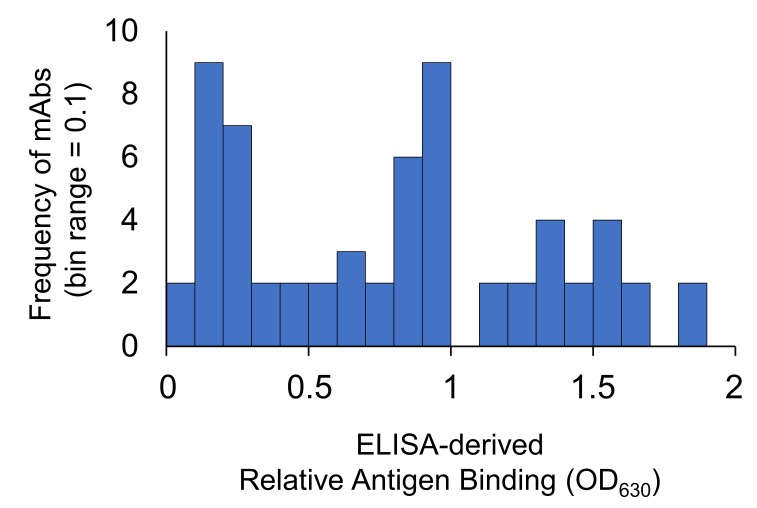
Histogram of relative antigen binding strength for the anti-LigB mAb libraries. Hybridoma supernatants containing individual mAbs were screened for efficient antigen binding using ELISA. The frequency of relative binding strength for both library C and V is depicted for each 0.1 OD_630_ range. The distribution can be grouped into populations of non-binding mAbs (0 < OD_630_< 0.5), weak binding mAbs (0.5 < OD_630_< 1.0), and moderate to high binding mAbs (1.0 < OD_630_< 2.0). Additional characterization was only pursued for the mAbs with moderate to high binding efficiencies. The division between weak and moderate binding efficiency also corresponded to the average between the lowest and highest OD_630_ for hybridoma supernatants in the ELISA screen.

### Binding properties of anti-LigB mAb libraries

**Figure 3.**
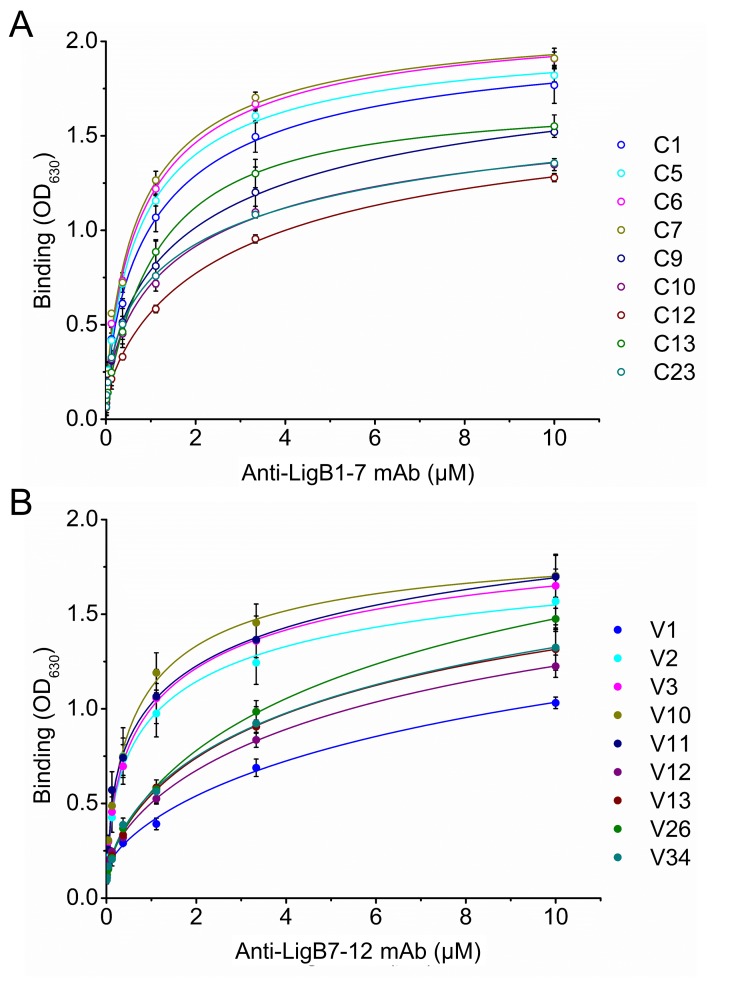
Equilibrium binding for anti-LigB mAbs. The equilibrium dissociation constants (K_D_) for mAbs from library C (**A**) and library V (**B**) were determined from dose-dependent binding curves. Increasing concentrations of purified anti-LigB mAbs (0.00686, 0.0137, 0.0412, 0.123, 0.370, 1.11, 3.33 and 10 µM) were incubated with LigB antigen (1 μM) immobilized on microtiter plates. The binding interaction was subsequently detected by ELISA using HRP-conjugated anti-mouse antibodies. All experiments were conducted in three trials, the mean ±S.D. of which were shown in bar charts.

For each library, nine mAbs were identified to have moderate to high binding efficiency to LigB antigens during the initial mAb screen. The dissociation constants (K_D_) for each of these mAbs was obtained from dose-dependent ELISA curves (Figure 3; Figure 3—figure supplement 1; Table 1). Based on K_D_ values, library C mAbs were generally able to bind tighter to the LigB antigen than library V mAbs. Several library C mAbs (C5, C6, C7) exhibit sub-micromolar K_D_ values while only one library V mAb (V10) was able to bind in the sub-micromolar range.

**Figure 3—figure supplement 1.**
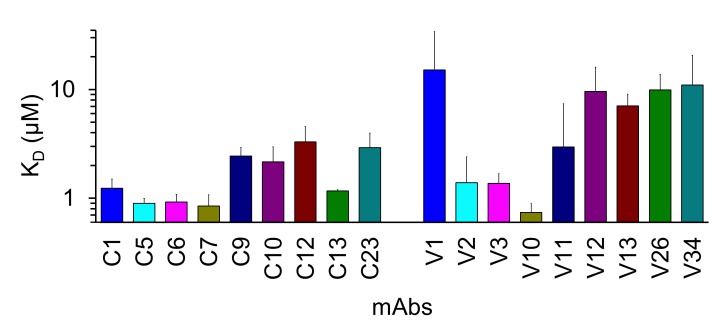
K_D_ values of anti-LigB mAbs. K_D_ values for library C (**A**) and library V (**B**) mAbs were calculated by fitting the ELISA-derived dose-dependent binding curves to a dose-response equation using Origin software. Errors for the K_D_ values obtained from nonlinear curve fitting are shown.

**Table 1.** Anti-LigB mAb characterization summary. Mean values for the dissociation constant (K_D_), FACS cell binding propensity (MFI), and lethal dose (LD_50_) are listed for mAbs against LigB1-7 (library C) and LigB7-12 (library V). Double and single domain specificities for mAbs are also noted.

**Anti-LigB mAbs**	K_D_ (μM)	Cell binding (MFI)	LD_50_ (μg/ml)	LigB domain specificity	
**Library C** **(Anti-LigB1-7)**				Double domain	Single domain
**C1**	1.232	3497.5	15.29	LigB1-2 LigB2-3	LigB2
**C5**	0.896	4499.5	15.07	LigB4-5	LigB5
**C6**	0.923	4644.5	13.76	LigB4-5	LigB5
**C7**	0.848	2722.5	18.72	LigB1-2	LigB1
**C9**	2.440	3530.0	19.56	LigB1-2	n.s.
**C10**	2.162		21.63	LigB4-5	n.s.
**C12**	3.301		11.57	LigB2-3 LigB4-5	LigB4 LigB6
**C13**	1.166	4186.0	18.98	LigB1-2 LigB4-5	n.s.
**C23**	2.916	3469.5	29.92	LigB2-3	n.s.
**C22**	n.b.	1121.5			
**Library V** **(Anti-LigB7-12)**				Double Domain	Single Domain
**V1**	15.151		25.85	LigB9-10 LigB10-11	LigB10
**V2**	1.391	2889.0	20.45	LigB11-12	LigB12
**V3**	1.367	2568.5	16.19	LigB7-8 LigB11-12	LigB7
**V10**	0.738	3211.0	14.87	LigB9-10 LigB10-11	LigB10
**V11**	2.956		19.24	LigB7-8 LigB11-12	LigB8 LigB12
**V12**	9.574	2333.5	29.18	LigB7-8	LigB7 LigB9
**V13**	7.064	2376.0	28.60	LigB7-8	LigB8
**V26**	9.915		22.83	LigB9-10 LigB10-11	n.s.
**V34**	11.011	2665.0	25.28	LigB7-8	LigB7 LigB9
**V33**	n.b.	910.5			
**Source of relevant data**	Figure 3, Figure 3—figure supplement 1	Figure 4B, Figure 4—figure supplement 2	Figure 5, Figure 5—figure supplement 1	Figure 4A, Figure 4—figure supplement 1	

n.b. no significant binding. n.s. no significant binding partner was determined.

**Figure 4.**
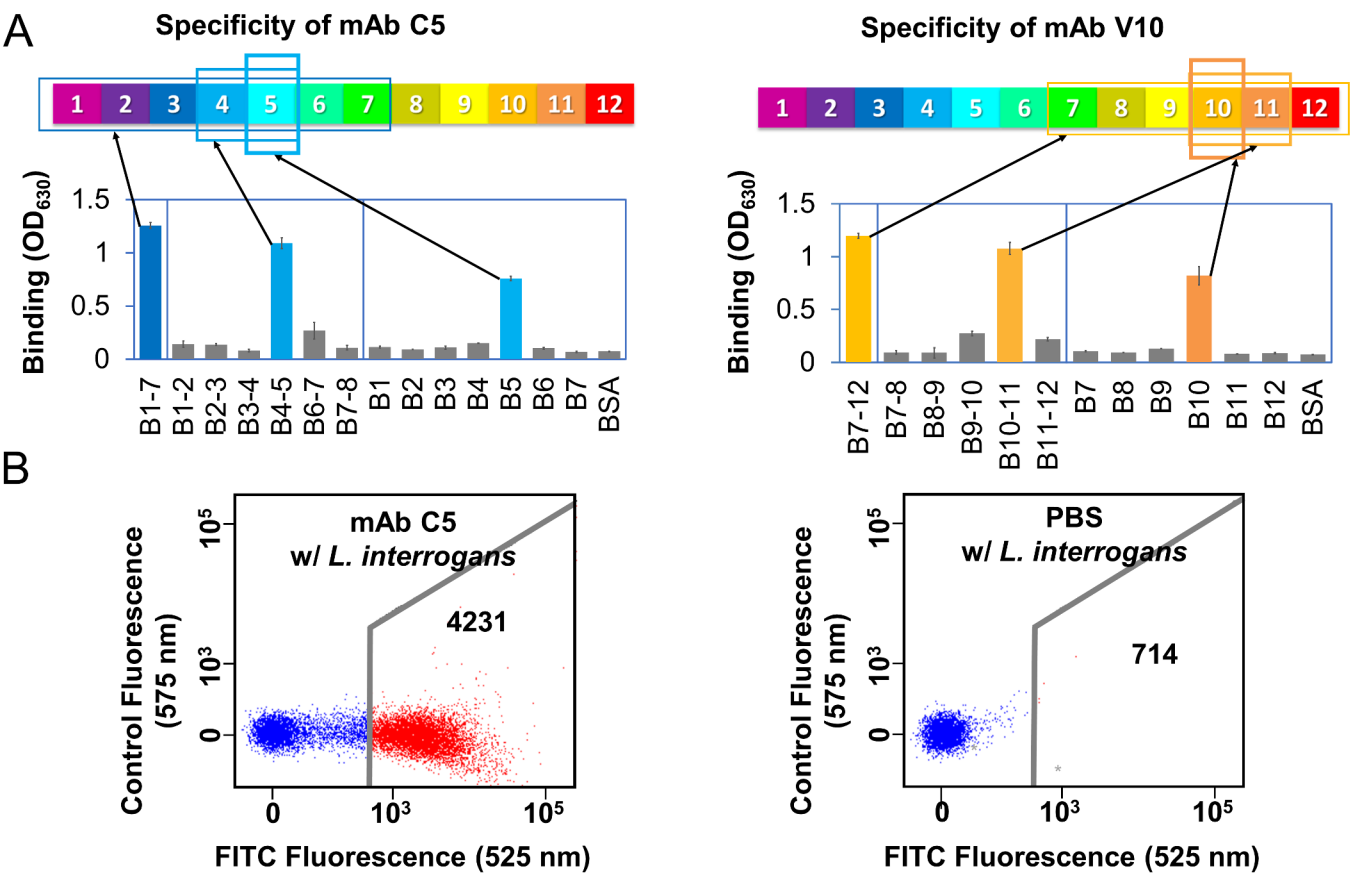
Anti-LigB mAb binding characterization. (**A**) A representative example of domain-level epitope ELISA-based mapping for anti-LigB mAbs (full dataset Figure 4—figure supplement 1). The mean ±S.D. for three trials is displayed. (**B**) Flow cytometry was used to measure the ability of anti-LigB mAbs to bind directly to live *Leptospira* (Figure 4—figure supplement 2B). The representative fluorescence emission displays the relative binding of fluorescently-labelled secondary anti-mouse IgG antibodies to PBS-treated cells (negative control, right panel) and to mAb C5-treated cells (left panel).

Domain-level specificity of individual LigB mAbs was investigated using a comprehensive set of single Ig-like domain LigB truncates and tandem (double) Ig-like domain LigB truncates (except LigB5-6) based on the NMR structure (Ptak et al., 2014) of the single domain, LigB12 (Figure 4—figure supplement 1). The LigB-derived antigens were immobilized on microtiter plates and tested for mAb binding specificity using an ELISA binding assay. The ELISA results for individual mAbs were generally able to localize binding to a specific double and/or single Ig-like domain. Figure 4A illustrates the ELISA assays used to identify the binding of mAbs C5 and V10 for specific two-domain regions and for specific single Ig-like domains. A comprehensive survey of domain-level epitope mapping for different mAbs are summarized in Table 1 and Figure 4—figure supplement 1. For the LigB1-7 antigen-derived library, immunoreactivity of mAbs was weighted towards LigB1-2 and LigB4-5 regions, while for the LigB7-12 antigen-derived library, mAbs were preferentially generated against LigB7-8 and LigB10-11 regions. Only 3 of 10 double domains, LigB3-4, LigB6-7, and LigB8-9, and 2 of 12 single domains, LigB3 and LigB11, lack immunogenicity for the set of tested mAbs. From an antigenic response to LigB1-7 and LigB7-12, at least every other individual Ig-like domain is capable of eliciting mAb production and the generated-LigB mAb libraries cover the length of the LigB Ig-like domain region. Several anti-LigB mAbs were tested using fluorescence-based flow cytometry for the ability to recognize native proteins on the surface of *Leptospira* cells. The spirochetes were incubated with mAbs from library C or V. Anti-LigB mAb-bound *Leptospira* cells were fluorescently-labelled with anti-mouse IgG antibodies and counted by flow cytometry. The strong fluorescence signal from incubation of the pathogenic *L. interrogans* serovar Pomona cells with anti-LigB mAb C5 was indicative of a tight cell surface interaction (Figure 4B). Cells incubated with either PBS (Figure 4B) or the negative control mAb C22 (Figure 4—figure supplement 2A) failed to generate a fluorescence signal after secondary anti-mouse IgG antibody incubation. Additionally, cells from *L. biflexa*, a non-infectious *Leptospira* species which lacks the genes for Lig proteins (Figueira et al., 2011), also failed to exhibit a fluorescence signal strong enough to indicate binding after anti-LigB mAb C5 incubation (Figure 4—figure supplement 2A). A total of seven library C mAbs and six library V mAbs were measured for *Leptospira* surface binding character and all produced a fluorescence signal count over three-fold higher than the PBS control and over two-fold higher than the poorly binding mAb controls (C22 and V33) (Figure 4—figure supplement 2B). The cell binding propensities for measured mAbs are listed as mean fluorescence intensity (MFI) values in Table 1. The flow cytometry data supports the presence of mAb-accessible Lig protein Ig-like domains on the surface of *Leptospira* cells.

**Figure 4—figure supplement 1.**
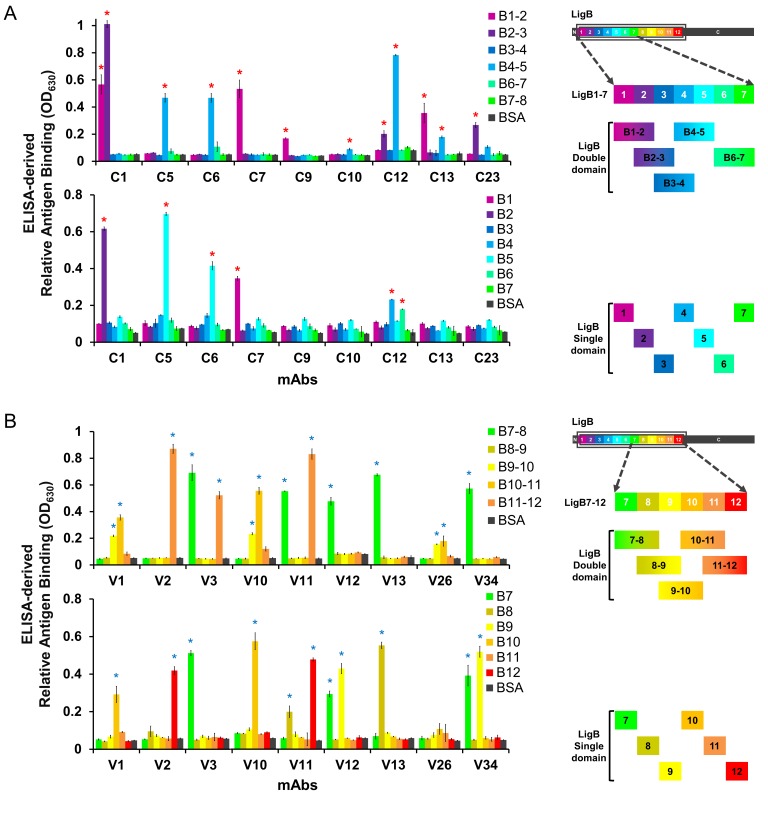
Domain-level epitope mapping of anti-LigB mAb library determined by ELISA. An array of double and single Ig-like domains from (**A**) LigB1-7 (1 μg/well) or (**B**) LigB7-12 (1 μg/well) and BSA (negative control) were coated on the microtiter plates for epitope mapping of mAbs. A fixed concentration (1 μM) of individual mAbs was applied to the LigB-coated wells. The binding levels of the mAbs were measured by rabbit anti-mouse IgG antibody conjugated with HRP (1:5000). Colors correspond to relative domain position. All experiments were conducted in three trials, the mean ±S.D. of which were shown in bar charts. Positive binding domains were identified by comparing with BSA and statistically significant (*t*-test; p<0.05) differences were marked by an asterisk and indicated in Table 1.

**Figure 4—figure supplement 2.**
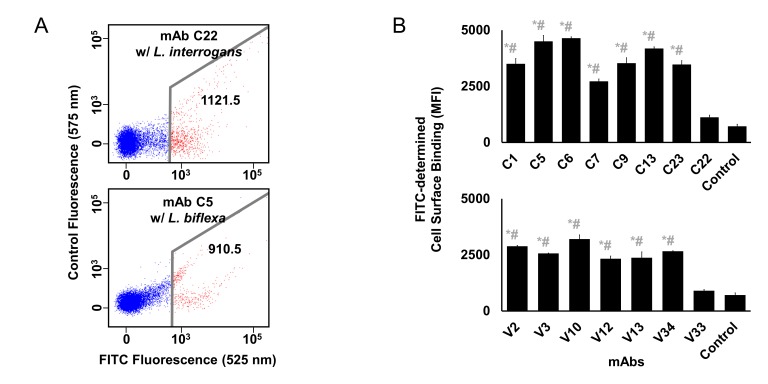
Cell surface binding of anti-LigB mAbs. (**A**) Flow cytometry was used to generate light scatter plots to obtain the mean fluorescence index (MFI) of FITC-positive cells from two negative control experiments (positive experiment displayed in Figure 4B). The mAb C22 (no binding to LigB1-7) binds poorly to live *Leptospira interrogans* Pomona (upper panel). The anti-LigB1-7 mAb C5 binds poorly to live non-pathogenic *Leptospira biflexa*, which does not express LigA or LigB proteins (lower panel). (**B**) The mean FITC-positive (MFI) count was obtained for a set of mAbs from libraries C and V. Each value represents the mean ± S.D. from two individual trials of two replicates. Statistically significant (*t*-test; p<0.05) differences were calculated from the comparisons between bacteria bound mAbs and corresponding weakly bound mAbs (*; C22, upper panel; V33, lower panel) or PBS control (#; both panels).

### Bactericidal activity of anti-LigB mAbs

**Figure 5.**
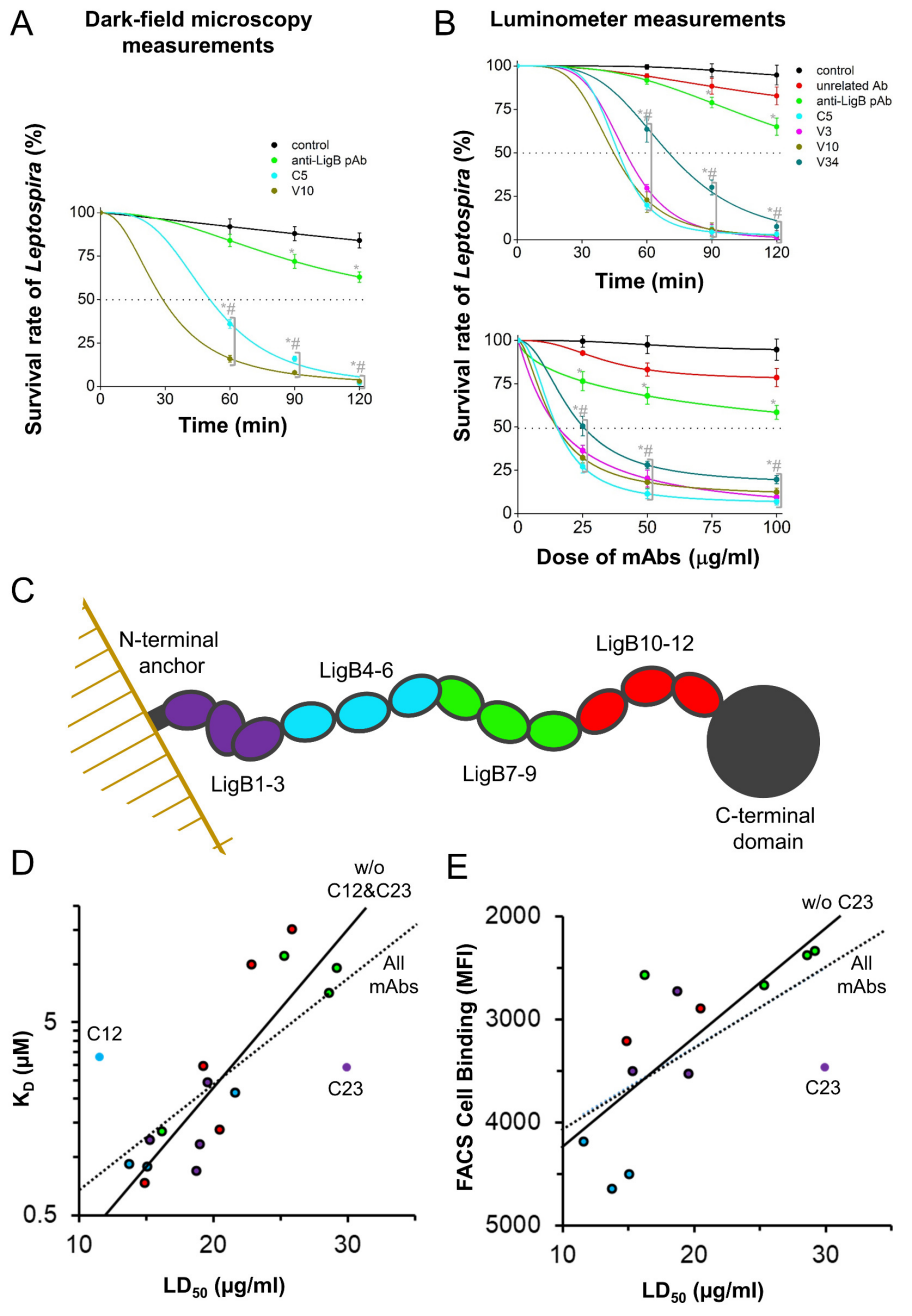
Anti-LigB mAb bactericidal properties. (**A, B**) *Leptospira* survival assays were used to assess mAb bactericidal activity. (**A**) After incubation with individual mAbs, the survival rate of *L. interrogans* Pomona was observed using dark-field microscopy (**B**) while the survival rate of bioluminance-producing *L. interrogans* Manilae was measured using a 96-well luminometer-based assay (full dataset Figure 5—figure supplement 1). Each value represents the mean ± S.D. from three individual trials of two replicates. Statistically significant (*t*-test; p<0.05) differences were calculated from the comparisons between mAb-treated groups and the control group (*) or between mAb-treated groups and the pAb group (#). (**C**) A schematic is shown for the SAXS-derived LigB structure. (**D**) For the set of mAbs, the ELISA antigen binding value is plotted against the LD_50_. Points for mAbs are colored to match the three domain regions defined in the schematic. Statistically-determined outliers are labelled and data was fit with and without outliers (dashed line, R²=0.508 and solid line, R²=0.773, respectively). (**E**) For mAbs measured for cell surface binding, the FACS cell binding value is plotted against the LD_50_ and data is fit with and without outlier, mAb C23 (dashed line, R²=0.312 and solid line, R²=0.466, respectively).

To test the role of the mAb libraries in promoting complement-mediated killing of *Leptospira in vitro*, the bactericidal activity of the LigB-binding mAbs was tested by incubating live *L. interrogans* serovar Pomona cells with complement-containing human serum with or without added antibodies. Using dark-field microscopy, motile bacteria were counted to calculate the survival rate at different time points. The PBS only (negative control) condition was unable to effectively kill the live *Leptospira* cells leaving an 84% survival rate at 120 min post treatment (Figure 5A). The hamster-derived polyclonal antibody (pAb) treatment of *Leptospira* cells led to an enhancement of bactericidal activity with only 63% survival at 120 min post treatment. *Leptospira* incubated with C5 or V10 mAbs showed only a 2% to 3% survival rate (a >20 fold improvement over pAb-incubated spirochetes p<0.05) (Figure 5A).

**Figure 5—figure supplement 1.**
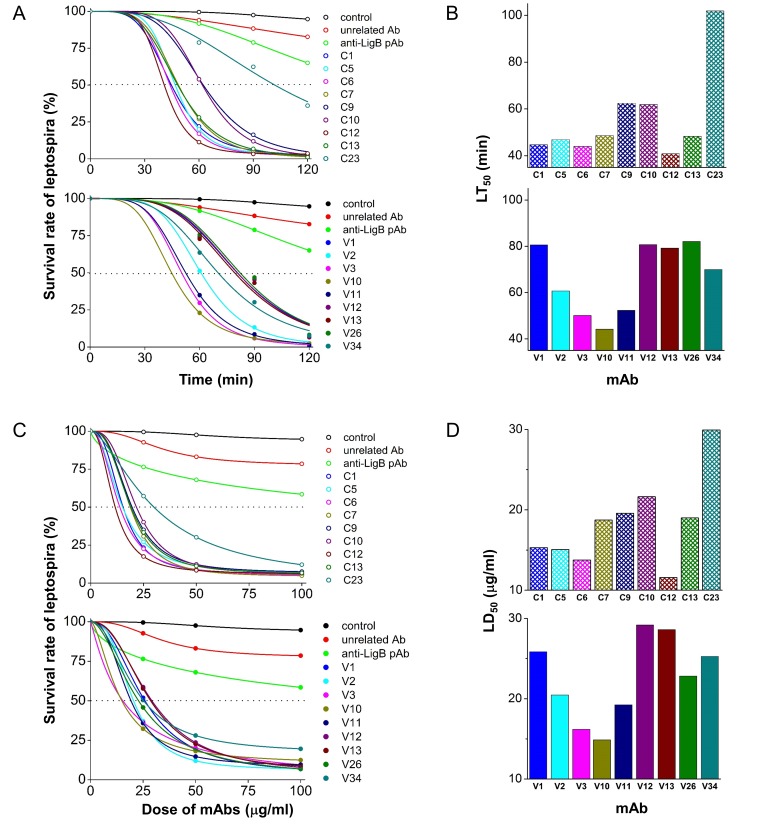
Serum bactericidal activity characterization of anti-LigB mAb library. (**A**) Time-dependence of mAb serum bactericidal activity. Bioluminance-producing *L. interrogans* Manilae were incubated with individual mAbs at 100 μg/ml. The leptospiral survival rates in the presence of mAbs and human serum complements were measured using a luminometer at different time points (60, 90, and 120 min). Negative controls were measured in the presence of no antibody (control) and an isotypic mouse IgG (unrelated Ab). Survival measurements for hamster anti-LigB polyclonal antibodies (anti-LigB pAb) are also shown. The data were fit using a standard logistic equation. (**B**) The median lethal time (LT_50_) was calculated for individual mAbs from the fitted logistic curve using Origin software. (**C**) Dose-dependence of mAb serum bactericidal activity. Bioluminance-producing *L. interrogans* Manilae were incubated with individual mAbs at various mAb concentrations (25, 50, and 100 μg/ml). The leptospiral survival rates in the presence of mAbs and human serum complements were measured at 90 min. The data were fit using a dose inhibition equation using Origin software. (**D**) The median lethal dose (LD_50_) was calculated for individual mAbs from the fitted dose inhibition curve.

**Figure 5—figure supplement 2.**
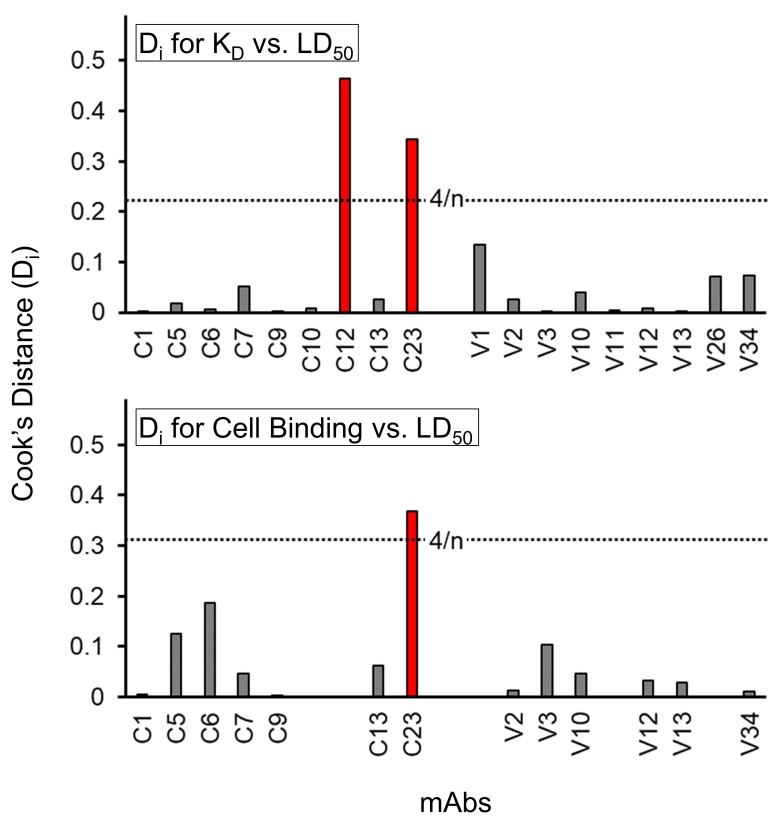
Statistically determined outliers for scatter plots. Cook’s distance (D_i_) demonstrates the influence of a point on a defined correlation. For the linear correlations of LOG(K_D_) vs. LD_50_ (Figure 5D) and FACS Cell Binding vs. LD_50_ (Figure 5E), D_i_ was determined for each data point. D_i_ values above the threshold of 4/n (red) deviate from the expected relationship between binding and bactericidal activity and were considered to be outliers.

**Figure 5—figure supplement 3.**
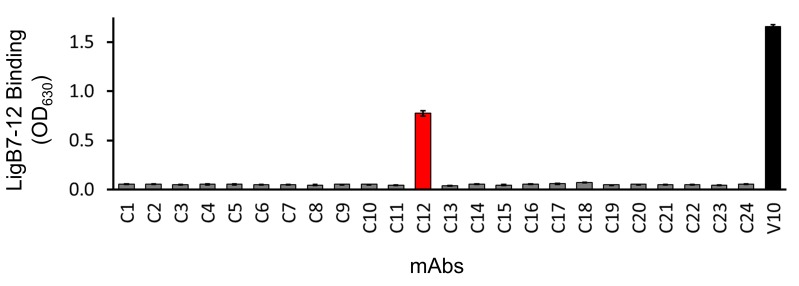
Binding of library C mAbs to LigB7-12. Library C mAbs were generated from hybridoma cell lines exposed to LigB1-7. The ability of library C mAbs to bind LigB7-12 was also assessed by colorimetic (λ_630_) TMB-ELISA. LigB7-12 binding was identified for C12 and V10 (positive control). Each value represents the mean ± S.D. from two individual trials of two replicates.

**Figure 5—figure supplement 4.**
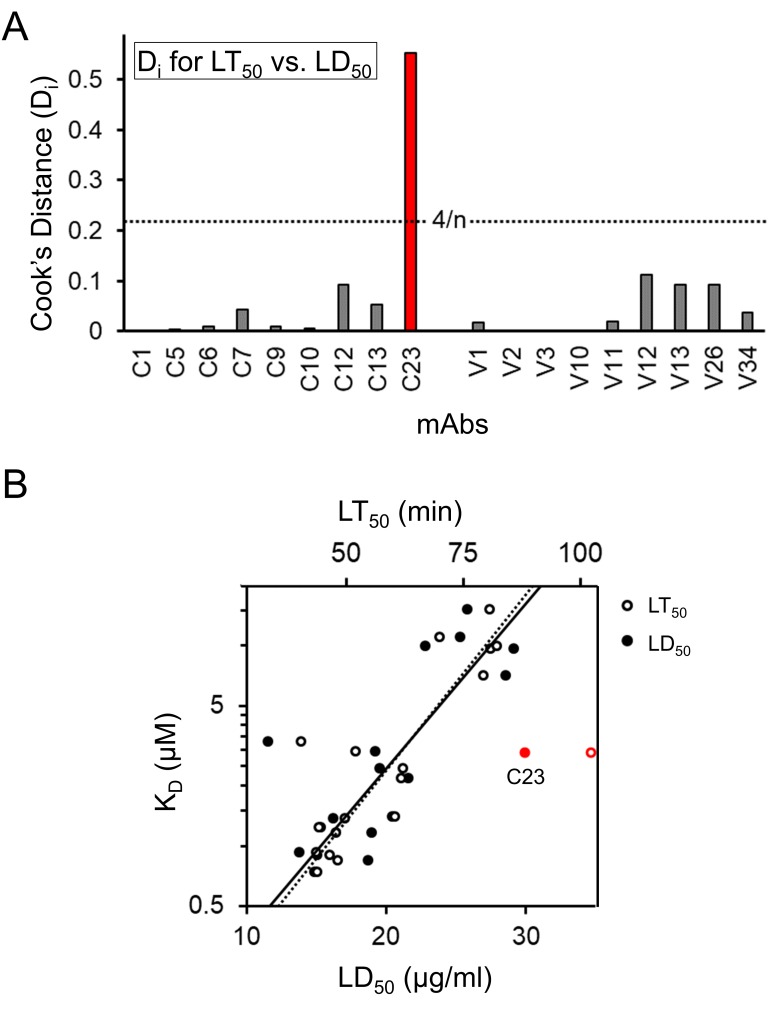
Comparison of bactericidal activity values, LT_50_ and LD_50_. (**A**) The Cook’s Distance (D_i_) was determined for the linear correlation of LT_50_ and LD_50_ (R^2^ = 0.867). C23 (red) was the only mAb with a D_i_ value above the outlier threshold. (**B**) Plots of K_D_ vs. LD_50_ and K_D_ vs. LT_50_ are overlaid and centered on fits with C12 and C23 removed (solid line: LD_50_R^2^ = 0.773, dashed line: LT_50_R^2^ = 0.872). The LT_50_ value of C23 (open red) is shifted farther away from the expected value than the LD_50_ value of C23 (solid red).

In addition, a high-throughput luciferase-based methodology was utilized to screen the bactericidal activity of the full set of mAbs. The assay measures the luminescence intensity from the expression of the light-generating *lux* cassette proteins in another pathogenic *L. interrogans* serovar Manilae to accurately report the viable cell count (Murray et al., 2010). Note that the LigB proteins from *L. interrogans* serovar Manilae and *L. interrogans* serovar Pomona share 96% identity suggesting that the bactericidal properties provided by the LigB (Pomona) mAb library using these two serovars would be similar. Bioluminescent *L. interrogans* serovar Manilae were again incubated with complement-containing human serum with or without added antibodies. The survival rate was determined by measuring the luminescence intensity of metabolically active *Leptospira* relative to that from the spirochetes prior to Ab-incubation. Both incubation time and antibody concentration were varied to obtain time-dependent and dose-dependent curves (Figure 5—figure supplement 1A and C, respectively) as well as LT_50_ and LD_50_ values (Table 1). Experimental analysis of luminometer measurements for control conditions and representative mAbs are presented in Figure 5B. The two negative controls, PBS only and non-specific mouse IgG1, as well as the hamster-derived pAbs were unable to kill >50% of cells at the longest time point (120 min) or at the highest dosage (100 µg/ml). All anti-LigB mAbs were able to eliminate >50% of *Leptospira* between 40 and 85 min with the exception of mAb C23 (Figure 5—figure supplement 1B). Each of the LigB-specific mAbs had the potential to kill >90% of the active *Leptospira* cells after a sufficient exposure time. All of the LigB-specific mAbs were able to effectively decrease the number of metabolically active *Leptospira* cells to less than 50% with a dose value (LD_50_) between 10 and 30 μg/ml (Figure 5—figure supplement 1D). The relative lack of efficiency of hamster pAbs compared to purified mAbs could be explained by the pAbs being a mixture of high affinity and low affinity antibodies, being a mixture of IgG subclasses with differing abilities to activate the complement system, or being derived from different rodents than the mAbs with potentially different abilities to activate the complement system. The ability of the anti-LigB mAbs to kill *Leptospira* in the presence of complement is consistent with their capability in binding to LigB protein in pathogenic *Leptospira*.

### Activity-Interaction map analysis of anti-LigB mAbs

In the context of a known antigen structure, the results of mAb-antigen interaction and mAb bactericidal activity experiments provide the opportunity to explore potential correlations between these three data sets. The broad mAb accessibility of LigB Ig-like domains supports the extended organization of the LigB1-12 SAXS structure. The LigB structure’s domain-domain arrangement is summarized in 3-domain segments (Figure 5C). The properties for each mAb have been correlated in plots of K_D_ vs. LD_50_ (Figure 5D) or FACS cell binding vs. LD_50_ (Figure 5E). Additionally, the mAb data points are colored to signify the mAb’s LigB segments binding specificity. A trendline for K_D_ vs. LD_50_ scatter plots using all data and excluding the two statistically-determined outliers (Figure 5D; Figure 5—figure supplement 2) yields correlations with R²=0.508 and R²=0.773, respectively. Antibodies specific to each of the four LigB segments are near the trendline. Thirteen anti-LigB mAbs were also tested for *Leptospira* surface binding ability. When plotted against LD_50_, FACS-derived cell binding values fit to a trendline with an R²=0.312 and the single outlier-adjusted trendline of with an R²=0.466 (Figure 5E; Figure 5—figure supplement 2). In both plots, near-trendline mAbs with specificity to the LigB1-3 and LigB4-6 segments have a relatively high binding ability and low LD_50_ and correlated mAbs with specificity to the LigB7-9 segment have a relatively low binding ability and high LD_50_.

Because mAbs that do not fit the predicted correlation between binding affinity and bactericidal activity have the potential to inform vaccine studies, the bases for outlier discrepancies were examined further. Only one outlier, C12, displays enhanced bactericidal activity above what would be expected based from binding correlations. While C12 displays some specificity for the straightest segment (LigB4-6), it can bind to two single domains (LigB4 and LigB6) as well as a double domain in the segment LigB1-3. Further, an additional ELISA assay found that C12 is the only library C mAb that can also bind to LigB7-12 (Figure 5—figure supplement 3). The ability of C12 to bind to multiple Ig-like domains covering a large range of the Ig-like domain regions suggests a reason for the increased bactericidal activity relative to C12’s binding affinity. The only outlier with bactericidal activity below expected is C23 which binds to LigB2-3. These domain neighbors exhibit the sharpest interdomain bend suggesting that steric limitations can differentially effect LigB binding and bactericidal activity. Indeed, the kinked structure of C23’s target domain may decrease the rate for C23 to initiate bacterial killing relative to its K_D_ (Figure 5—figure supplement 4). Overall, the link between antigen binding and bactericidal activity complements the highly accessible extended structure of LigB with highly accessible regions having the potential to yield high bactericidal mAbs. The high degree of correlation between antigen-mAb binding and bactericidal activity supports the hypothesis that a mAb’s ability to kill a pathogen can be determined by its antigen-specific binding affinity.

### LigB Ig-like domain chimeras identify epitopes on each side of the 3-D fold

**Figure 6.**
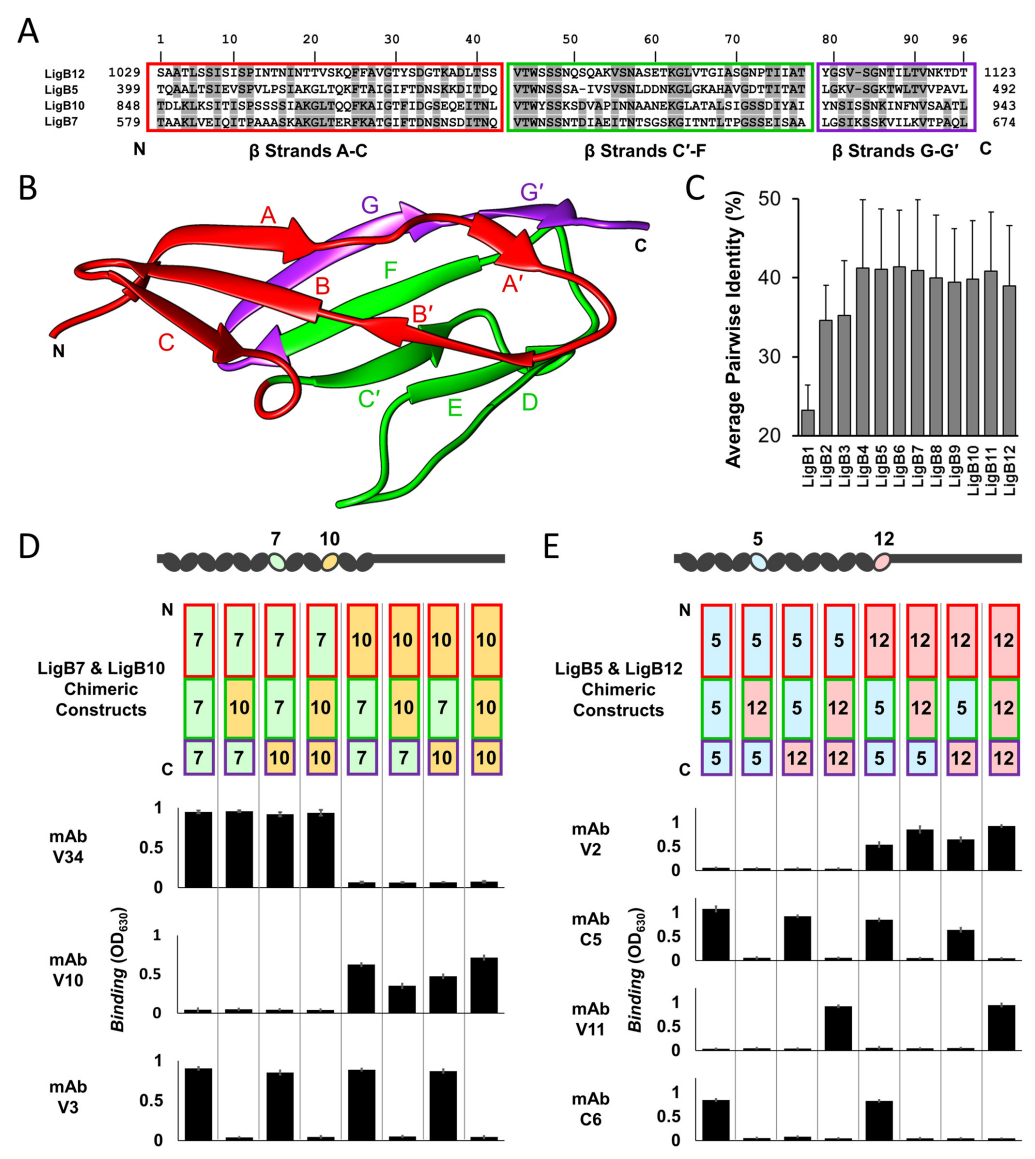
Dissection of mAb binding surfaces using chimeras. (**A**) Sequence alignment of LigB Ig-like domains used for generating chimeras. The chimeric swap regions are divided into colored boxes. Residues that are identical in either LigB5 and LigB12 or LigB7 and LigB10 pairs are shaded. (**B**) The structure of LigB12 (PDB ID 2MOG) is correspondingly colored by chimeric swap regions. (**C**) Average percent identity ± S.D. for each LigB Ig-like domain based on a pairwise matrix. (**D**) The set of LigB7 and LigB10 single domain chimeric constructs which cover all variations for the three swapped regions (schematic representation shown) were tested for binding to mAbs with LigB7-binding specificity (V34 and V3) or LigB10-binding specificity (V10) using an ELISA assay. (**E**) LigB5 and LigB12 chimeras were similarly tested for binding to mAbs with LigB5-binding specificity (C5 and C6) or LigB12-binding specificity (V2 and V11). The ELISA binding values correspond to the chimeric construct representation at the top of the column. All ELISA experiments were conducted in three trials, the mean ± S.D. of which were shown in bar charts.

To provide proof that a single Ig-like domain can act as a scaffold to display multiple protective epitopes, the binding epitope for anti-LigB mAbs was more specifically mapped to a surface within individual Ig-like domains. Chimeric LigB Ig-like domains were designed to differentiate interactions specific to the surface residues of major folding units. The high degree of homology between LigB Ig-like domains (Figure 6A and C) provided an opportunity for tertiary structural elements to be exchanged at two chimeric swapping positions (Ptak et al., 2014) (Figure 6A and B). The first two segments of the chimeras (β-strands A-C and β-strands C′-F) were engineered to separate the top and bottom halves of the Ig-like domain β-sandwich. The third chimera region (β-strands G-G′) was included to determine if an antigenic surface is formed on the untethered edge of the β-sandwich. Four single domain proteins that have been recognized to contain mAb-specific epitopes (Table 1) were paired based on matching length to generate two sets of chimeras (LigB5/LigB12 and LigB7/LigB10). Including the wild-type domains, eight possible chimeric combinations were generated on single Ig-like domains (Figure 6D and E).

**Figure 6—figure supplement 1.**
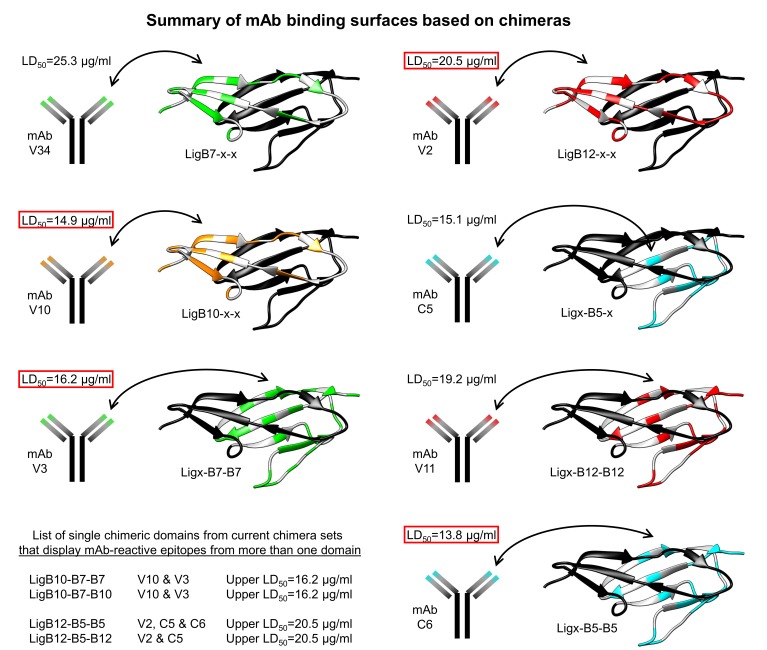
Summary of mAb binding surfaces based on chimeras. LigB Ig-like domain chimeras were used to screen which protein surfaces possessed immunoreactivity to the library of LigB mAbs. The Ig-like domain region of mAb specificity is mapped to the corresponding residue location of the LigB12 structure (PDB ID 2MOG). Colored residues (green, LigB7; orange, LigB10; red, LigB12; cyan, LigB5) indicate the location of non-identical residues within the chimera set (LigB7/B10 or LigB5/B12), while white residues indicate identical residues in the chimera parent alignments. The mAb LD_50_ (Table 1) is listed with the lowest value specific to the N-terminal sheet (strands A-C) and the C-terminal sheet (strands C′-G′) for each chimera set boxed in red. The upper LD_50_ is also listed (lower left) for chimeric domains that can be detected with dual-parent domain mAb specificity.

All mAbs showed binding to a subset of the eight proteins with a clear region-specific pattern (Figure 6D and E). Two LigB7-specific mAbs (V12 and V34), two LigB10-specific mAbs (V1 and V10), and one LigB12-specific mAb (V2) could bind only to the N-terminal β-strands A-C from their respective interacting domains. The LigB5-specific mAb C5 and LigB7-specfic mAb V3 interacted with proteins that contained β-strands C′-F from LigB5 and LigB7, respectively. Unique to the LigB5/LigB12 mAb set, mAb V11 and mAb C6 required both the β-strands C′-F and the terminal β-strands G-G′ from LigB12 and LigB5, respectively. The mAb binding patterns demonstrate that the surface contribution of both β-strands A-C and β-strands C′-F (or C′-G′) can provide distinct epitopes for direct mAb targeting.

### Chimera LigB10-B7-B7 confers enhanced protection against *Leptospira* lethal challenge

**Figure 7.**
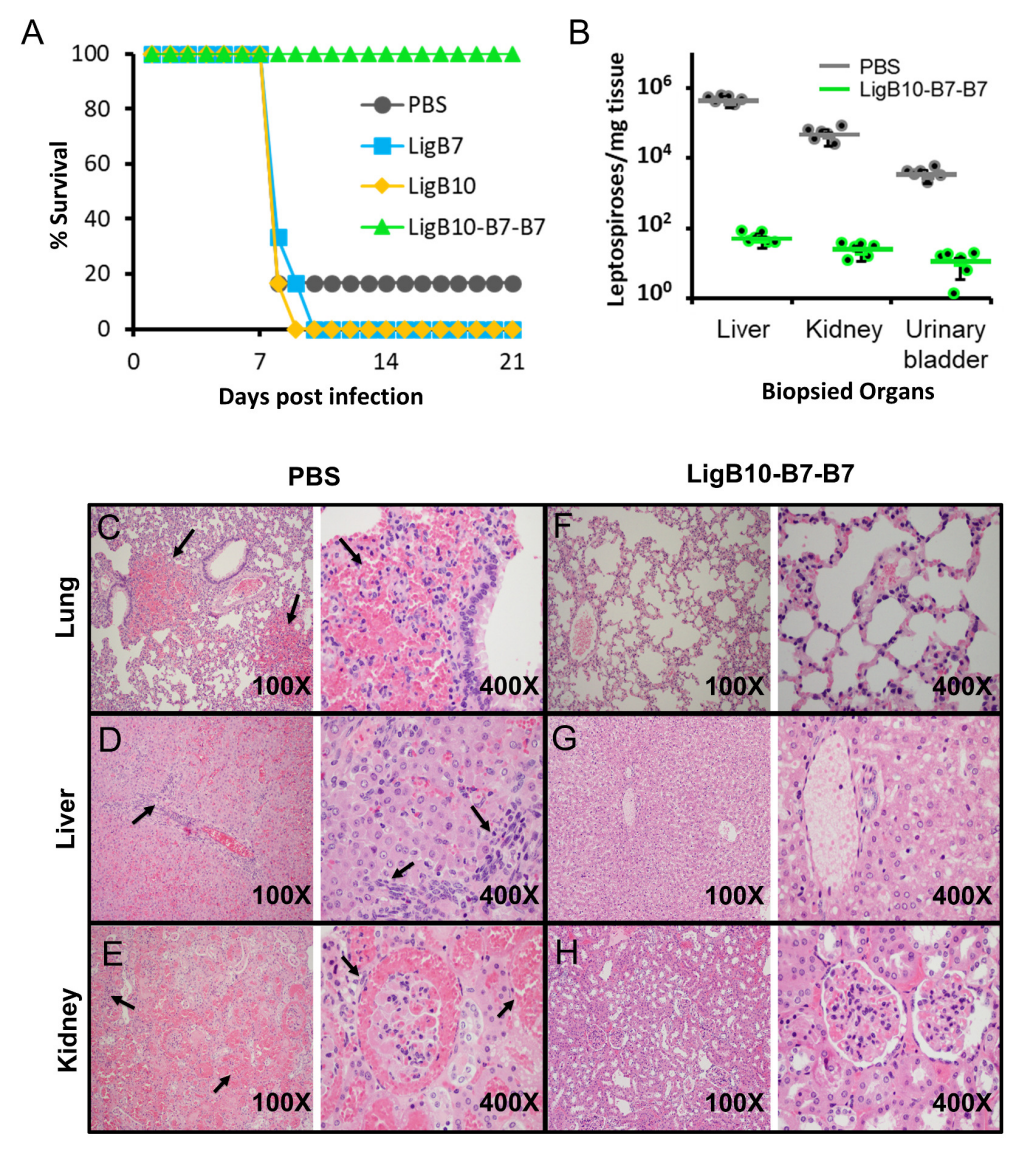
*Leptospira* lethal challenge of hamsters immunized with LigB Ig-like domains. (**A**) Survival rates for groups of six immunized hamsters are shown for the 3 weeks post leptospiral challenge. Inoculations and boosters with specific LigB Ig-like domains occurred 3 and 6 weeks prior to leptospiral infection. Sera from immunized hamsters were tested for domain-specific immunoreactivity (Figure 7—figure supplement 2). (**B**) The indicated organ tissues were biopsied from post-challenged hamsters immunized with PBS (control) or LigB10-B7-B7. The leptospiral load of hamster tissue was determined by RT-qPCR for the *Leptospira* specific gene, LipL32. Each point depicts the mean value obtained from duplicate analysis of individual tissue samples. Bars indicate the mean bacterial loads ± S.D. (six hamsters per group). Leptospiral loads for the PBS group are significantly higher than those of the LigB10-B7-B7 group in all three tissues. (C-H) Post-challenged hamster tissues that are targets for spirochete infection were fixed by formalin and stained by hematoxylin and eosin. PBS (control) immunized hamster tissue exhibited (**C**) multifocal lung hemorrhage, (**D**) liver inflammation and necrosis, and (**E**) tubulointerstitial nephritis and hemorrhage in the kidneys (each indicated by arrows). (**F-H**) Tissue from the LigB10-B7-B7 immunized group were all within normal limits.

The ultimate goal of combining structural and immunoreactivity studies of antigens is to explore a strategy for the rational engineering of improved vaccines. A chimeric LigB Ig-like domain was evaluated for the potential to elicit an immune response to multiple regions of LigB and to thereby enhance vaccine protection against leptospiral infection. The chimera LigB10-B7-B7 (β-strands A-C: B10 and β-strands C′-F and G-G′: B7) was chosen as the best candidate for animal studies because the chimeric domain possesses the ability to bind highly bactericidal mAbs specific to each parent domain (Figure 6—figure supplement 1). Additionally, LigB10-B7-B7 was similar to wild-type LigB7 and LigB10 in protein expression levels and in overall secondary structure (circular dichroism analysis, Figure 7—figure supplement 1). To generate a protective response, hamsters were immunized with 50 µg of LigB7, LigB10, LigB10-B7-B7, or PBS (as a negative control) for two times at 3 week intervals, and then challenged with 2.5 × 10^2^ of triple passages of *L. interrogans* Pomona (Conrad et al., 2017; Kunjantarachot, 2014) (Figure 7A). On day 8, the leptospiral challenge was lethal for five of six hamsters inoculated with PBS group, three of five hamsters inoculated with wild-type LigB7, and four of five hamsters inoculated with wild-type LigB10. By day 10, all of the wild-type LigB7 and LigB10 immunized hamsters had either died or were euthanized due to severe clinical signs. In contrast, all five of the hamsters that had been inoculated with LigB10-B7-B7 and subsequently challenged with *Leptospira* survived until the end of the experiment (day 21 post infection). The survival rate of the LigB10-B7-B7 group is significantly higher (100%) than either control group (17%) or individual wild-type domain groups (0%) (p<0.05).

**Figure 7—figure supplement 1.**
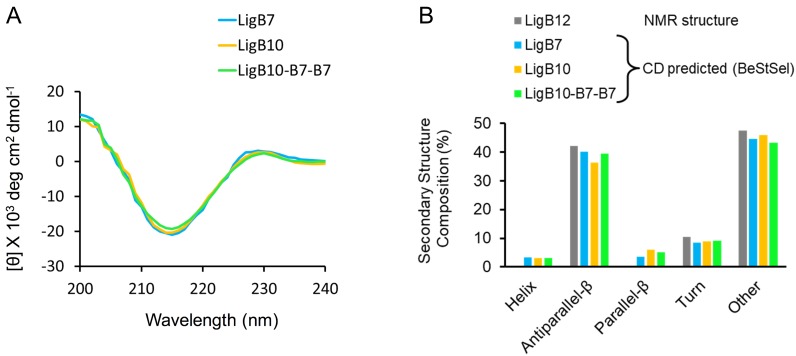
Secondary structure analysis for LigB Ig-like domains utilized in immunization trials. (**A**) Far-UV circular dichroism (CD) spectra were measured for LigB7, LigB10, and LigB10-B7-B7. The molar ellipticity, θ, was measured from 200 nm to 240 nm for 10 μM of each protein at room temperature. Mean values from three independent measurements were used to generate the spectra. (**B**) Using the experimental CD spectra, the secondary structure compositions for LigB7, LigB10, and LigB10-B7-B7 were predicted with the BeStSel webserver. The top Class, Architecture, and Topology predictions for all three LigB proteins were Mainly Beta, Sandwich, and Immunoglobulin-like, respectively. The BeStSel-derived secondary structure composition of LigB12 obtained from the NMR structure (PDB ID 2MOG) is reported for comparison.

**Figure 7—figure supplement 2.**
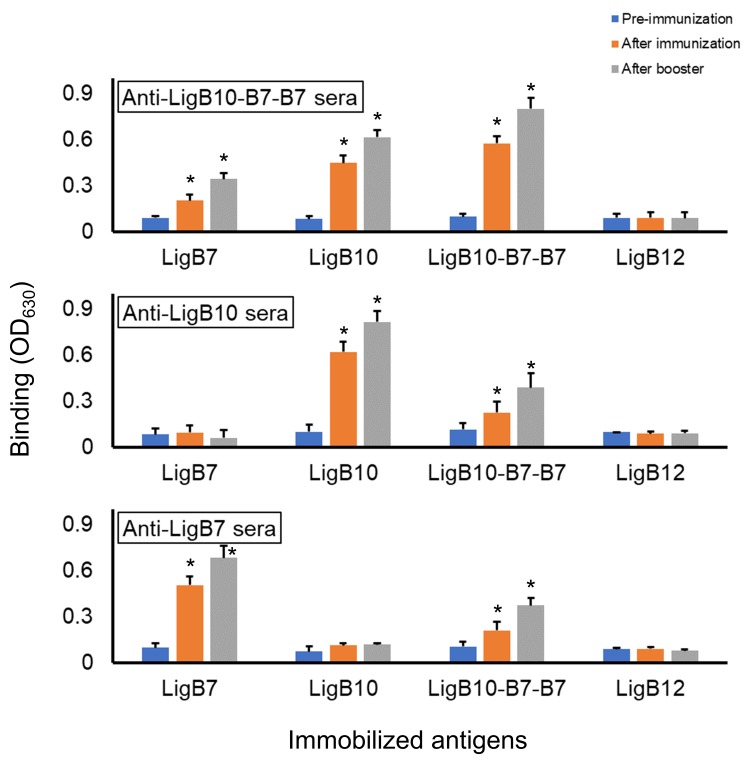
Detection of humoral response. The serological fluid of hamsters immunized with recombinant LigB Ig-like domain vaccines showed strong antibody generation against the corresponding domain antigens in an ELISA assay (orange, after immunization; gray, after booster). Anti-LigB10 and Anti-LigB7 sera exhibited immunoreactivity against LigB10-B7-B7 but not LigB12 (negative control) while Anti-LigB10-B7-B7 sera exhibited immunoreactivity against LigB10 and LigB7 but not LigB12 (negative control). All experiments were conducted in three trials, the mean ± S.D. of which were shown in bar charts. Positive sera immunoreactivities were identified by comparing with LigB12 and statistically significant (*t*-test; p<0.05) differences were marked by an asterisk.

**Figure 7—figure supplement 3.**
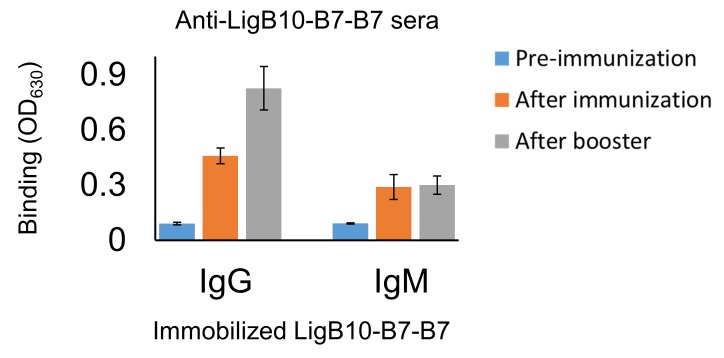
Memory response triggered by LigB10-7-7 chimera. The serological fluid of hamsters immunized with recombinant LigB10-7-7 antigen was collected before immunization, after immunization (primary response), and after the booster (secondary response). Anti-LigB10-B7-B7 sera was applied to recombinant LigB10-7-7 coated microtiter plates and tested for antigen-specific IgG or IgM using secondary antibodies (anti-hamster IgG or IgM conjugated with HRP). All experiments were conducted in three trials, the mean ± S.D. of which were shown in bar charts.

**Figure 7—figure supplement 4.**
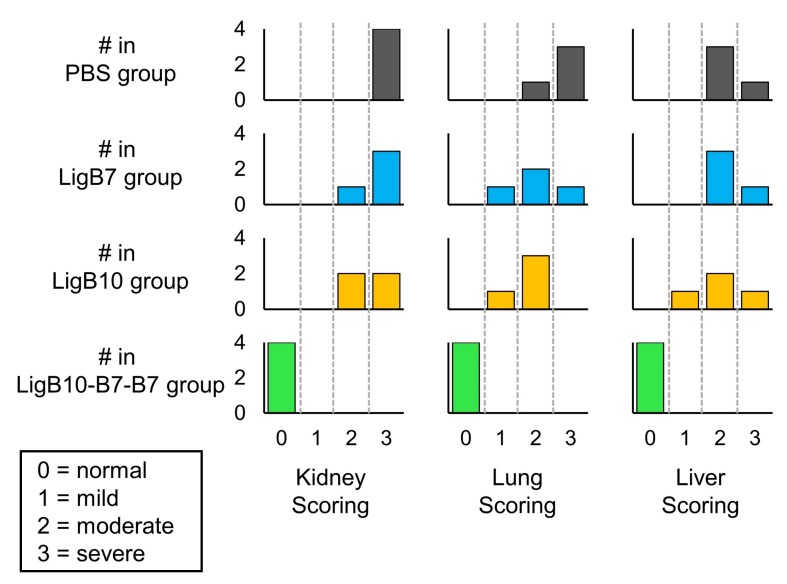
Histograms of post-challenged hamster tissue scores for PBS, LigB7, LigB10, and LigB10-7-7 inoculated groups. Respective tissues from four hamsters in each immunization group were individually harvested and fixed. Histopathological scoring was performed by a board certificated veterinary pathologist. Kidney tissues were graded for severity of renal lesions (0 = normal, 1 = mild, 2 = moderate, 3 = severe tubulointerstital nephritis). Lung tissues were graded for severity of hemorrhage (0 = normal, 1 = focal, 2 = multifocal, 3 = locally extensive areas of hemorrhage). Liver tissues were graded for the number of inflammatory foci (0 = normal, 1 = 1–3, 2 = 4–7, 3 =>7).

Serological fluids collected during the hamster studies were tested for the ability of the immunizations to generate an effective humoral response using a direct ELISA binding assay. The sera from LigB domain immunized hamsters provided a strong antibody response against the corresponding immobilized recombinant LigB domain (Figure 7—figure supplement 2). LigB7, LigB10, and LigB10-B7-B7 boosters were able to further enhance the antibody response. The Anti-LigB10-B7-B7 sera was also reactive with wild-type LigB7 and LigB10 but failed to react with the negative control, LigB12. LigB10-B7-B7 immunization led to an increase in post-booster secondary response for IgG but not IgM antibodies implying the generation of a typical immune memory response (Figure 7—figure supplement 3). The serological tests indicate that targeted antibodies can be effectively generated by vaccines based on individual LigB Ig-like domains and chimeric domains.

Several complementary methods were employed to test for the vaccine’s ability to reduce bacterial burdens of host tissue. Liver, kidney, and urinary bladder tissue from all immunization groups were examined by real-time quantitative reverse transcription polymerase chain reaction (RT-qPCR) to identify the *Leptospira* specific gene, LipL32 (Figure 7B) (Levett et al., 2005; Haake et al., 2000). The hamster group that received immunizations containing recombinant chimeric protein, LigB10-B7-B7, exhibited a significant reduction in leptospiral burden when compared to bacterial loads from wild-type LigB7 or LigB10 immunized hamsters. The immunized hamsters were further examined for histopathologic changes of livers, kidneys and lungs (Figure 7C–H and Figure 7—figure supplement 4). Except for one apparently normal hamster from PBS group, all other hamsters from PBS, LigB7 and LigB10 immunization groups presented severe clinical signs and prominent macroscopic lesions on spirochete-targeted organs (e.g. multifocal pulmonary ecchymoses, icteric liver and enlarged kidney). Lung lesions included thickening of alveolar septa due to edema, interstitial leukocytes infiltration, endothelial cell swelling, and extensive hemorrhage (Figure 7C). Leptospira-infected livers were infiltrated by various inflammatory cells, indicating moderate to severe hepatitis. Focal necrosis was also found in parenchymal hepatocytes, leading to loss of normal tissue integrity (Figure 7D). Severe tubulointerstitial nephritis with locally extensive hemorrhage in uriniferous spaces and tubules was present in Leptospira-infected kidneys (Figure 7E). Furthermore, greater than 50% of renal tubules were lost and replaced by lymphoplasmacytic cells infiltration and severe fibrosis. In contrast, all hamsters immunized with chimeric LigB10-B7-B7 presented no visible macroscopic/microscopic lesions in livers, kidneys or lungs (Figure 7F–H). In agreement with high survival rate of LigB10-B7-B7 immunized hamsters, vaccines containing the chimeric antigen provide superior protection and potentially sterilizing immunity for the animals from leptospiral infection.

## Discussion

*Leptospira*, like other pathogenic bacteria, have evolved a wide variety of disease-related surface proteins to initiate colonization, to combat host defense systems and to reach target organs based on tissue-specific tropism (Ko et al., 2009; Vieira et al., 2014). Lig proteins contribute to pathogenesis by acting as both surface adhesins to mediate attachment to host extracellular matrix (ECM) molecules (Lin et al., 2009a; Vieira et al., 2014; Figueira et al., 2011) and recruiters of complement regulators to evade attack by the host’s innate immune system (Breda et al., 2015; Choy et al., 2007; Hsieh et al., 2016; Castiblanco-Valencia et al., 2012). In addition, Ig-like domains from LigB are currently some of the strongest recombinant protein-based vaccine candidates in protecting hamsters from lethal challenges although discrepancies in effectiveness suggest that vaccine redesign efforts could help to create an improved vaccine (Cao et al., 2011; Conrad et al., 2017; Yan et al., 2009; Adler, 2015). This study provides new information regarding the architecture and accessibility of the main host-interacting region of LigB. The elongated structure presents insight into the mechanism of Lig protein adhesion, but, more importantly, an analysis of the mAb exposed regions and lack of long range inter-domain interactions has provided guidance for the optimization of recombinant leptospirosis vaccines using homologous Lig protein domains.

The domain arrangement of the LigB1-12 Ig-like domain region was determined using low resolution SAXS structures. The SAXS-derived LigB1-12 structure provides a visual depiction that complements the more therapeutically applicable information from mAb accessibility. The domain accessibility predicted by the LigB architecture also suggested that including the full set of Ig-like domains as antigens for mAb library generation was likely to be worthwhile. While previous SAXS studies have generated composite multi-domain structures (Jeffries et al., 2011; Morgan et al., 2012), the full LigB structure is the first example of using a sliding window of medium length, 5-domain constructs to generate a much longer 12-domain structure. The advantage of using a sliding window is that the high level of redundancy within the 5-domain structures allows for an increased accuracy in orienting and positioning the segments relative to each other. The sequence of domains LigB1-6 is identical to LigA domains 1–6 so the SAXS structure provides a partial structure of the LigA Ig-like domain region. While LigA is not present in all pathogenic *Leptospira* species, LigA-based vaccines, like LigB-based vaccines, enhance immunity to leptospirosis (Palaniappan et al., 2006; Silva et al., 2007; Faisal et al., 2009).

Recombinant antigen-based vaccine technologies are the most promising avenue for the development of optimized protective strategies against leptospirosis. In recombinant antigen design, the basic antigen scaffold must present a native and well-folded epitope to the immune system while a minimized antigen can eliminate nonessential, disruptive features (Kulp and Schief, 2013). Examples of vaccines against different variants of *Neisseria meningitides* and Lyme disease *Borreliae* have been generated from chimeric recombinant antigens and have been capable of inducing broad spectrum bactericidal mAbs (Earnhart and Marconi, 2007; Scarselli et al., 2011). Ig-like domains are found in a variety of prokayotic and eukaryotic extracellular proteins and readily fold to their native structure (Bodelón et al., 2013). By incorporating various epitopes onto a homologous scaffold, the overall structural integrity of the antigenic surface can be maintained on the chimeric LigB Ig-like domain construct. Based on the high resolution LigB12 structure, each of the three chimeric segments encompassed a 900 to 2000 Å^2^ surface and could potentially make a full conformational epitope (Ptak et al., 2014). While only two of three LigB chimeric segments were able to present as a full epitope experimentally, the third segment was required for binding by several of the mAbs. Indeed, initial experiments suggest that a redesigned chimera can harbor three separate epitopes on a single LigB Ig-like domain scaffold. Interestingly, the single domain LigB10-B7-B7 provides better protection to hamsters than two longer constructs, LigB7-12 (which provided poor protection) and LigB1-7 (which provided good protection) (Yan et al., 2009; Conrad et al., 2017). Exposure of epitopes in single domain antigens which would otherwise be blocked by host factors (Beernink et al., 2011) (i.e., ECM or serum proteins [Lin et al., 2009a; Breda et al., 2015; Hsieh et al., 2016]) in multi-domain constructs may enhance the efficiency. Two future goals of LigB recombinant vaccine studies will be to optimize further the chimeric antigens, consciously limiting the functionality of host-interacting sites, and to offer cross-species protection against different serovars.

This study and other recent studies (Ptak et al., 2014; Conrad et al., 2017) have significantly advanced our understanding of Lig protein structure and the potential of recombinant vaccines. The field of leptospirosis is poised to take advantage of the new insights and could make significant improvements in vaccines and other treatments to reduce the agricultural and human impact of the pathogen, *Leptospira*.

## Materials and methods

**Key resources table:** 

Reagent type (species) or resource	Designation	Source or reference	Identifiers	Additional information
gene (*Leptospira interrogans* serovar Pomona)	LigB (1-12)	GenBank	GenBank:FJ030916	
recombinant DNA reagent	pET28-His-Sumo (plasmid)	other		Re-engineered by Dr. Mao's lab (Manford et al., 2010)
strain, strain background (*Escherichia coli*)	*E. coli* Rosetta strain	Novagen	Novagen:70954	
commercial assay or kit	Ni-NTA resin	Qiagen		
peptide, recombinant protein	Sumo protease (Ulp-1)	other		protein expression by Dr. Chang's lab utilizing pET28-His- Sumo vector provided by Dr. Mao's lab, and IPTG induction in Rosetta strain *E. coli*
commercial assay or kit	Superdex75 (size exclusion chromatography)	GE Healthcare	GE_Life_Sciences: 17517401	
software, algorithm	BeStSel	DOI:10.1073/pnas.1500851112		Uses CD spectra obtained from Aviv-201 spectropolarimeter
software, algorithm	Clustal Omega	DOI:10.1093/nar/gkt376	RRID:SCR_001591	
software, algorithm	ATSAS Suite (GNOM; DAMMAVER; DAMMIF; AMBIMETER)	DOI:10.1107/S1600576717007786	RRID:SCR_015648	
software, algorithm	RAW	DOI:10.1107/S1600576717011438		
biological sample (*L. interrogans*)	LigB1-7	this lab		protein expression utilizing pET28-His- Sumo vector and IPTG induction in Rosetta strain *E. coli*
biological sample (*L. interrogans*)	LigB7-12	this lab		protein expression utilizing pET28-His- Sumo vector and IPTG induction in Rosetta strain *E. coli*
biological sample (*L. interrogans*)	LigB5-12	this lab		protein expression utilizing pET28-His- Sumo vector and IPTG induction in Rosetta strain *E. coli*
commercial assay or kit	Protein A/G Chromatography	ThermoFisher Scientific Pierce		
cell line (*Mus musculus*)	hybridoma clones	William Davis Laboratory		mAb-producing cell lines generated from BALB/c mice immunized with recombinant LigB antigen
antibody	HRP-conjugated anti-mouse IgG (goat polyclonal)	Invitrogen	RRID:AB_2533947	(1:5000)
antibody	anti-LigB1-7 (mouse monoclonal)	this lab		(1:500)
antibody	anti-LigB7-12 (mouse monoclonal)	this lab		(1:500)
antibody	HRP-conjugated anti-hamster IgG goat polyclonal)	KPL	https://www.seracare.com/products/kpl-antibodies-and-conjugates/secondary-antibodies/anti-hamster-igg--h-l--antibody/	(1:1000)
antibody	HRP-conjugated anti-hamster IgM goat polyclonal)	SouthernBiotech	https://www.southernbiotech.com/?catno=6060-05&type=Polyclonal	(1:1000)
antibody	FITC-conjugated anti-mouse IgG (goat polyclonal)	ThermoFisher Scientific	RRID:AB_2533946	(1:1000)
antibody	isotypic control IgG (mouse Fc fragment)	ThermoFisher Scientific	RRID:AB_10959891	
strain, strain background (*Leptospira biflex*)	*Leptospira biflex*	other		Colony maintained in Dr. Chang's lab
strain, strain background (*L. interrogans*)	*Leptospira interrogans* serovar Pomona	this lab		Colony maintained in Dr. Chang's lab
strain, strain background (*L. interrogans*)	*Leptospira interrogans* serovar Manilae M1307	other		Colony maintained in Dr. Gerald Murray and Dr. Ben Adler's labs, provided as a gift
biological sample (*Homo sapiens sapiens*)	Normal human serum	ImmunoReagents	ImmunoReagents: SP-001-VX10	
biological sample (*Mesocricetus auratus*)	Golden Syrian Hamster	Harlan Sprague Dawley Laboratory		5 weeks old at initial subcutaneous vaccination
chemical compound, drug	Adjuvant 2% Alhydrogel	InvivoGen		

### Cloning, protein expression and purification

A series of single (LigB1 to LigB12), double (LigB1-2 to LigB11-12, excluding LigB5-6), five-domain (LigB1-5 to LigB8-12), and multiple (LigB1-7, LigB7-12, and LigB5-12) Ig-like domains of LigB from *Leptospira interrogans* serovar Pomona (GenBank, FJ030916) (illustrated in Figure 1—figure supplement 1A, Figure 2—figure supplement 1A, and Figure 4—figure supplement 1) were constructed on the vector pET28-His-Sumo between BamHI and HindIII (or XhoI) restriction enzyme sites as previously described (Manford et al., 2010; Lin et al., 2009b; Lin et al., 2009a). The constructed plasmids containing LigB genes were transformed to *E. coli* Rosetta strain and the protein expression was induced with 1 mM isopropyl β-D-thiogalactopyranoside (IPTG) at 20 ˚C for 16 hr. After the cells were lysed using a high-pressure cell disruptor, the cell lysates were spun down and the supernatants were purified by Ni-NTA resin (Qiagen). The His-Sumo tagged LigB proteins were eluted with phosphate buffered saline (PBS; 137 mM sodium chloride, 10 mM sodium phosphate, 2.7 mM potassium chloride, 1.8 mM potassium phosphate) containing 300 mM imidazole and then digested with Sumo protease Ulp-1 while dialyzing against PBS buffer at 4 ˚C overnight (Manford et al., 2010). Afterwards, the digested proteins were applied to a second Ni-NTA resin to remove the His-Sumo tag, and the untagged proteins were collected in the flow-through fraction. To gain higher protein purity, the untagged Lig proteins were further separated from other contaminates by size exclusion chromatography (SEC) Superdex75 (GE Healthcare), resulting in only one major species migrating on SDS-PAGE. For secondary structure analysis, circular dichroism (CD) spectra of LigB proteins were measured on an Aviv 202–01 spectropolarimeter (Aviv Biomedical, Lakewood, NJ) and predicted secondary structure composition was obtained using BeStSel (Micsonai et al., 2015). Chimeric LigB5/LigB12 and LigB7/LigB10 constructs were created by overlapping extension PCR to produce all six possible swapped genes from three protein segments (Figure 6). The three segments, strand A-C, strand C′-F and strand G-G′, and specific residue boundaries were identified based on the high-resolution NMR structure of LigB12 (PDB ID 2MOG) (Ptak et al., 2014) and the sequence alignment of parent domains. The percent identity matrix for the set of Ig-like domains was obtained using the EMBL-EBI web service Clustal Omega (McWilliam et al., 2013). After ligation into pET28-Sumo vectors, the selected positive constructs were isolated from kanamycin resistant colonies and confirmed by DNA sequencing.

### SAXS construct window optimization

Constructs corresponding to two, five and eight domains were generated to identify the optimal domain number required to obtain useful SAXS-derived envelopes (Figure 1—figure supplement 1). The 8-domain long construct (LigB5-12) failed to generate envelopes with distinct domain regions representative of possible 8-domain structures. High average normalized spatial discrepancy (NSD) values for a reconstruction indicate increased variability among the trial models used to build the final envelope. Increasing average NSD with longer constructs may reflect a wider range of conformations expected with longer chains but additional factors could influence NSD statistics for longer constructs. The maximum diameter of a model is limited by the minimum q value measured in the scattering profile, as dictated by the Shannon Limit (Putnam et al., 2007). Thus, for these experiments, q_min = 0.1 implies that models can be no longer than pi/0.1 = 314 Å, under ideal conditions. In reality, minor aggregation and noise at low q may also place additional limits on model length. Mylonas and Svergun have also shown that reconstructions of long rods are not as reliable as more compact structures (Mylonas and Svergun, 2007). For these reasons, we have chosen to assemble a full-length model from shorter overlapping constructs. SAXS envelopes derived from two or five sequential domains were generally comprised of distinct domain regions and accurately reflected the number and size of expressed domains. Of the 2- and 5-domain structures, only the envelopes of five sequential domains provided the relative orientation of multiple neighboring domains.

### Small angle X-ray scattering

LigB-derived proteins were exchanged into PBS buffer (pH 7.4) and concentrated to 12–20 mg/ml as assessed by absorption at UV_280_. SAXS data for individual samples was measured for 15 scans at 1×, 2/3×, and 1/3 × protein concentrations after dilution with PBS buffer and centrifugation at 14,000 rpm for 10 min. Capillary cells were robotically loaded with 30 μL samples from a 96 well-plate maintained at 4°C (Nielsen et al., 2012). Between each sample, the capillary cell was thoroughly washed with detergent and water and then dried with air. All SAXS experiments were collected at the Cornell High-Energy Synchrotron Source (CHESS)’s F2 or G1 beamline using a dual Pilatus 100 K-S SAXS/WAXS detector (Acerbo et al., 2015; Skou et al., 2014). Background subtraction of SAXS buffer and further data and statistical analysis were performed using the free open-source software, RAW (Nielsen et al., 2012; Hopkins et al., 2017). The GNOM program from the ATSAS suite was used to determine P(*r*) plots. Optimal Rmax was determined by screening at 5 Å intervals and the P(*r*) plots were normalized to the first peak. Profiles best representing the dilute (ideal) solution limit were used to generate *ab initio* models with DAMMAVER and DAMMIF programs from the ATSAS suite (Petoukhov et al., 2012; Volkov and Svergun, 2003). 10 initial models were used to calculate the final model. Models with a normalized spatial distribution > mean + 2*standard deviation were treated as outliers and not included in determining the final model. No more than one model was excluded for each structure. Model uniqueness was evaluated using the AMBIMETER program (Petoukhov and Svergun, 2015). AMBIMETER score ranges < 1.5 indicate a unique ab initio shape determination, 1.5–2.5 indicate some potential for alternate solutions, and >2.5 indicate that multiple shape solutions will fit the data.

### Monoclonal antibody (mAbs) production

A 5 mg total of LigB1-7, and separately 5 mg of LigB7-12, was used to immunize and boost the antibody production from five BALB/c mice. Hybridoma clone generation was conducted in the laboratory of Dr. William Davis (fee-for-service) as previously described (Park et al., 2015). Standard ELISA assays were used for in-house screening of the hybridoma clones for positive supernatants. A total of 24 clones of IgG-type mAbs against LigB1-7 (library C; Lig protein Conserved region) and 36 clones of IgG-type mAbs against LigB7-12 (library V; Lig protein Variable region) were generated. Each library produced 9 mAbs which were found to be of moderate to high binding efficiency and utilized for further characterization. Purification of mAbs was conducted by protein A/G chromatography (Pierce) using the manufacturer-recommended procedure with minor modification (Eliasson et al., 1989). Briefly, the hybridoma supernatant was dialyzed against the binding buffer (100 mM potassium phosphate, 150 mM sodium chloride, pH 8.0) overnight at 4 ˚C. The dialyzed sample was applied to a protein A/G column (pre-equilibrated with binding buffer). After washing with 10–15 ml of binding buffer to remove the unbound fraction, the bound mAb was eluted with 100 mM glycine at pH 3.0 and neutralized immediately with 1 M Tris at pH 9.0. The eluate containing mAb was dialyzed against PBS buffer at pH 7.0, concentrated to 3–4 mg/ml, and stored at −80 ˚C.

### ELISA binding assay

LigB-binding mAbs were selected using an ELISA assay (Lin et al., 2009a; Lin et al., 2011). Microtiter plates (Nunc MaxiSorp, ThermoFisher Scientific) were coated with 1 μg of LigB1-7 or LigB7-12 in coating buffer (0.2 M NaHCO_3_, pH 9.4) at 4˚C overnight. After blocking with 3% BSA in PBS buffer for 1 hr, hybridoma supernatants were prepared at 1/500 dilutions and individually applied to LigB-coated wells for initial screening. To obtain dissociate constants, selected mAbs with moderate to high binding affinity to LigB were serially diluted in PBS (0.00686, 0.0137, 0.0412, 0.123, 0.370, 1.11, 3.33 and 10 µM) and then individually applied to LigB-coated wells. Between each step, PBS containing 0.05% Tween-20 (PBS-T) was used to wash the plates for three times. Subsequently, anti-mouse IgG antibody conjugated with HRP (1:5000, Invitrogen) was added to detect the binding of mAbs presented in hybridoma supernatants. Finally, 100 μl of TMB peroxidase substrate (KPL) was applied to each well, the optical density of which was recorded at 630 nm by ELx808 Absorbance Microplate Reader (BioTek). OD_630_ values represent the mean of three independent trials ± the standard deviation. For each trial, samples were assayed in two replicates.

For examining the domain specificities of mAbs, single domain LigB fragments (LigB1 to LigB12), double domain LigB fragments (LigB1-2 to LigB11-12, excluding LigB5-6), positive control (LigB1-7, LigB7-12), or negative control (BSA) were coated with a fixed concentration (1 μg/well) on the ELISA plates. Each mAb against LigB1-7 or LigB7-12 was then added to corresponding single-domain or double-domain LigB coated wells. For binding analysis of each chimera, the set of six chimeric LigB proteins plus the two parent LigB proteins were coated on the ELISA plates (1 μg/well). Each mAb with activity against either of the two parent LigB proteins was screened for binding activity on the LigB coated wells. For measuring the antibody responses triggered by LigB-based recombinant vaccines, sera from hamsters immunized with LigB7, LigB10 and LigB10-7-7 were collected at different time points (pre-immunization, post-immunization and post-booster). Then, these serum samples (1:500) were added to corresponding LigB Ig-like domain coated wells (1 μg/well), and also applied to LigB12 coated wells (negative control). Finally, anti-hamster IgG or IgM antibody conjugated with HRP (1:1000, KPL or SouthernBiotech) was used to detect the LigB-bound antibodies from hamster sera. All single and double domain ELISA experiments including single chimeric domain experiments were conducted in three trials, the mean ±S.D. of which were shown in bar charts.

### Surface binding of mAbs to live leptospira by flow cytometry

*Leptospira interrogans* freshly harvested from hamsters challenged with serovar Pomona or overnight NaCl-treated low passage *Leptospira* were prepared in PBS buffer containing 5 mM MgCl_2_ at 10^8^ cells/ml. Various anti-LigB mAbs (100 µg/ml) were individually applied to the bacterial suspension at 1:500 dilution. After a 1 hr incubation at room temperature, the bacteria-mAbs mixtures were spun down at 2,000 g for 7 min and then washed with PBS containing 1% BSA. Subsequently, goat anti-mouse antibodies conjugated with fluorescein (FITC) (1:1000) were used as secondary antibodies for probing the bacteria-bound mAbs. After another PBS-BSA wash, the bacteria-mAbs mixtures were fixed by 0.5% formaldehyde in PBS. The non-pathogenic *Leptospira biflex*, which does not express LigB, was subjected to the same procedure by incubating with selected mAbs for the control experiments (Figueira et al., 2011). Another negative control was conducted by treating the pathogenic *Leptospira* with unrelated mouse IgG (isotypic mouse IgG purchased from ThermoFisher Scientific). Flow cytometry was performed at the Cornell University Flow Cytometry Core facility using a BD LSR-II (BD Biosciences) instrument with the excitation laser at 488 nm and the emission wavelength at 525/575 nm. Unstained *Leptospira* was identified by forward scatter (FSC) and side scatter (SSC). Selected mAb (C5) treated *L. interrogans* without secondary antibodies (FITC-conjugated goat anti-mouse IgG) was used as a negative control to set the gating region. The mean FITC-positive (MFI) count was obtained for the gated region of the 525 nm vs. 575 nm scatter plots using BD FACSDiva software. For each sample, at least 20,000 cells were analyzed in two independent trial of two replicates.

### Serum bactericidal assay

To examine if the mAbs were effective at killing bacteria in vitro, the serum bactericidal activity (SBA) assay was conducted. 10^8^ cells/ml of low passage, high virulent *L. interrogans* seorvar Pomona and Manilae strain M1307 were prepared in PBS buffer containing 2 mM MgCl_2_ and 1 mM CaCl_2_, and then mixed with respective mAbs, plus 25% of normal human serum (ImmunoReagents) as a complement source. The mixtures were incubated at 37°C for 1 hr, and the viability of the bacteria were examined using dark-field microscopy. The survival rate of leptospira was calculated as the number of motile (alive) cells in every 100 counts separately by two researchers (blind). The mean value was obtained from the two independent (blind) measurements as a single technical replicate. Three independent trials of two replicates were measured.

The viability of the leptospira was also accessed from the luminescence emitted by metabolically active *L. interrogans* Manilae strain M1307 (Murray et al., 2010). The same preparations were examined at different time points (0, 30, 60 and 90 min), and the luminescence intensity of each sample was measured by GloMax 96 Microplate Luminometer (Promega). The survival rate of leptospira was calculated by the intensity at a specific time point divided by the intensity at 0 min. In addition, a series of dilutions of mAbs (100 μg/ml to 3.125 μg/ml) were evaluated for dose dependent bactericidal efficacy of antibodies. The time (LT_50_) and dose (LD_50_) required to reach 50% lethality were obtained from fitted logistic and dose inhibition curves, respectively (Origin 7.0).

### Hamster challenge

Hamsters (Harlan Sprague Dawley Laboratory) were housed in isolation units approved by the Cornell University Institutional Animal Care and Use Committee (Protocol number: 2015–0133). The Golden Syrian Hamsters used in vaccine trials were allowed to run free in the cage, were fed a commercial ration, and were provided water *ad libitum* as previously described (Kunjantarachot, 2014). Six 5-week-old hamsters each were vaccinated subcutaneously for each LigB-based recombinant vaccine containing adjuvant 2% Alhydrogel (InvivoGen) at 3 week intervals for a total of two injections. The control group was injected with adjuvant only. Three weeks after the final vaccination, all animals were challenged with 2.5 × 10^2^ of triple passaged *L. interrogans* seorvar Pomona through the intraperitoneal route as previously described (Kunjantarachot, 2014). Kidneys, livers, lungs and urinary bladders were biopsied from hamsters within 1 hr after euthanasia (Palaniappan et al., 2006). Leptospiral loads found in livers, kidneys and urinary bladders from all immunization groups were examined by real-time RT-qPCR (Levett et al., 2005). The total RNA was extracted from target tissues and then reverse transcribed to cDNA. Subsequently, the *Leptospira* specific gene, LipL32 (Haake et al., 2000), was amplified and detected with fluorescence by 7500 Fast Real-Time PCR system. Histopathological tissue slices were fixed with 10% neutral buffered formalin and stained with hematoxylin and eosin. Tissue samples were imaged and scored by light microscopy.

## Additional files

Transparent reporting form

### Major datasets

The following previously published datasets were used:

Chang YF2008FJ030916: Leptospira interrogans serovar Pomona isolate pLPLIGB LigB (ligB) gene, partial cds.http://www.ncbi.nlm.nih.gov/nuccore/FJ030916Publicly available at NCBI Nucleotide (accession no. FJ030916)

Ptak CPHsieh CLin YMaltsev ASRamanRSharma YOswald REChang Y2014Solution structure of the terminal Ig-like domain from Leptospira interrogans LigB.http://www.rcsb.org/pdb/explore/explore.do?structureId=2MOGPublicly available at the RCSB Protein Data Bank (accession no. 2MOG)

## References

[bib1] Acerbo AS, Cook MJ, Gillilan RE (2015). Upgrade of MacCHESS facility for X-ray scattering of biological macromolecules in solution. Journal of Synchrotron Radiation.

[bib2] Adler B (2015). Vaccines against leptospirosis. Current Topics in Microbiology and Immunology.

[bib3] Beernink PT, Shaughnessy J, Braga EM, Liu Q, Rice PA, Ram S, Granoff DM (2011). A meningococcal factor H binding protein mutant that eliminates factor H binding enhances protective antibody responses to vaccination. The Journal of Immunology.

[bib4] Bodelón G, Palomino C, Fernández LÁ (2013). Immunoglobulin domains in Escherichia coli and other enterobacteria: from pathogenesis to applications in antibody technologies. FEMS Microbiology Reviews.

[bib5] Breda LC, Hsieh CL, Castiblanco Valencia MM, da Silva LB, Barbosa AS, Blom AM, Chang YF, Isaac L (2015). Fine mapping of the interaction between C4b-Binding protein and outer membrane proteins LigA and LigB of Pathogenic Leptospira interrogans. PLOS Neglected Tropical Diseases.

[bib6] Cao Y, Faisal SM, Yan W, Chang YC, McDonough SP, Zhang N, Akey BL, Chang YF (2011). Evaluation of novel fusion proteins derived from extracellular matrix binding domains of LigB as vaccine candidates against leptospirosis in a hamster model. Vaccine.

[bib7] Castiblanco-Valencia MM, Fraga TR, Silva LB, Monaris D, Abreu PA, Strobel S, Józsi M, Isaac L, Barbosa AS (2012). Leptospiral immunoglobulin-like proteins interact with human complement regulators factor H, FHL-1, FHR-1, and C4BP. The Journal of Infectious Diseases.

[bib8] Chang YF, Chen CS, Palaniappan RU, He H, McDonough SP, Barr SC, Yan W, Faisal SM, Pan MJ, Chang CF (2007). Immunogenicity of the recombinant leptospiral putative outer membrane proteins as vaccine candidates. Vaccine.

[bib9] Choy HA, Kelley MM, Chen TL, Møller AK, Matsunaga J, Haake DA (2007). Physiological osmotic induction of Leptospira interrogans adhesion: LigA and LigB bind extracellular matrix proteins and fibrinogen. Infection and Immunity.

[bib10] Choy HA (2012). Multiple activities of LigB potentiate virulence of Leptospira interrogans: inhibition of alternative and classical pathways of complement. PLoS ONE.

[bib11] Conrad NL, Cruz McBride FW, Souza JD, Silveira MM, Félix S, Mendonça KS, Santos CS, Athanazio DA, Medeiros MA, Reis MG, Dellagostin OA, McBride AJ (2017). LigB subunit vaccine confers sterile immunity against challenge in the hamster model of leptospirosis. PLOS Neglected Tropical Diseases.

[bib12] Correia BE, Bates JT, Loomis RJ, Baneyx G, Carrico C, Jardine JG, Rupert P, Correnti C, Kalyuzhniy O, Vittal V, Connell MJ, Stevens E, Schroeter A, Chen M, Macpherson S, Serra AM, Adachi Y, Holmes MA, Li Y, Klevit RE, Graham BS, Wyatt RT, Baker D, Strong RK, Crowe JE, Johnson PR, Schief WR (2014). Proof of principle for epitope-focused vaccine design. Nature.

[bib13] Costa F, Hagan JE, Calcagno J, Kane M, Torgerson P, Martinez-Silveira MS, Stein C, Abela-Ridder B, Ko AI (2015). Global morbidity and mortality of Leptospirosis: A systematic review. PLOS Neglected Tropical Diseases.

[bib14] Dormitzer PR, Ulmer JB, Rappuoli R (2008). Structure-based antigen design: a strategy for next generation vaccines. Trends in Biotechnology.

[bib15] Dormitzer PR, Grandi G, Rappuoli R (2012). Structural vaccinology starts to deliver. Nature Reviews Microbiology.

[bib16] Earnhart CG, Marconi RT (2007). An octavalent lyme disease vaccine induces antibodies that recognize all incorporated OspC type-specific sequences. Human Vaccines.

[bib17] Eliasson M, Andersson R, Olsson A, Wigzell H, Uhlén M (1989). Differential IgG-binding characteristics of Staphylococcal protein-A, Staphylococcal protein-G, and a chimeric protein AG. Journal of Immunology.

[bib18] Faisal SM, Yan W, McDonough SP, Chang YF (2009). Leptospira immunoglobulin-like protein A variable region (LigAvar) incorporated in liposomes and PLGA microspheres produces a robust immune response correlating to protective immunity. Vaccine.

[bib19] Figueira CP, Croda J, Choy HA, Haake DA, Reis MG, Ko AI, Picardeau M (2011). Heterologous expression of pathogen-specific genes ligA and ligB in the saprophyte Leptospira biflexa confers enhanced adhesion to cultured cells and fibronectin. BMC Microbiology.

[bib20] Grassmann AA, Souza JD, McBride AJ (2017). A Universal Vaccine against Leptospirosis: Are We Going in the Right Direction?. Frontiers in Immunology.

[bib21] Haake DA, Chao G, Zuerner RL, Barnett JK, Barnett D, Mazel M, Matsunaga J, Levett PN, Bolin CA (2000). The leptospiral major outer membrane protein LipL32 is a lipoprotein expressed during mammalian infection. Infection and Immunity.

[bib22] Hopkins JB, Gillilan RE, Skou S (2017). BioXTAS RAW: improvements to a free open-source program for small-angle X-ray scattering data reduction and analysis. Journal of Applied Crystallography.

[bib23] Hsieh CL, Chang E, Tseng A, Ptak C, Wu LC, Su CL, McDonough SP, Lin YP, Chang YF (2016). Leptospira immunoglobulin-like protein B (LigB) binds to both the C-terminal 23 amino acids of fibrinogen αC domain and factor XIII: insight into the mechanism of LigB-mediated blockage of fibrinogen α chain cross-linking. PLOS Neglected Tropical Diseases.

[bib24] Jeffries CM, Lu Y, Hynson RM, Taylor JE, Ballesteros M, Kwan AH, Trewhella J (2011). Human cardiac myosin binding protein C: structural flexibility within an extended modular architecture. Journal of Molecular Biology.

[bib25] Ko AI, Goarant C, Picardeau M (2009). Leptospira: the dawn of the molecular genetics era for an emerging zoonotic pathogen. Nature Reviews Microbiology.

[bib26] Kulp DW, Schief WR (2013). Advances in structure-based vaccine design. Current Opinion in Virology.

[bib27] Kunjantarachot A (2014). Immunogenicity of Leptospira interrogans Outer Membrane Vesicles in a Hamster Model. Journal of Vaccines & Vaccination.

[bib28] Lessa-Aquino C, Lindow JC, Randall A, Wunder E, Pablo J, Nakajima R, Jasinskas A, Cruz JS, Damião AO, Nery N, Ribeiro GS, Costa F, Hagan JE, Reis MG, Ko AI, Medeiros MA, Felgner PL (2017). Distinct antibody responses of patients with mild and severe leptospirosis determined by whole proteome microarray analysis. PLOS Neglected Tropical Diseases.

[bib29] Levett PN, Morey RE, Galloway RL, Turner DE, Steigerwalt AG, Mayer LW (2005). Detection of pathogenic leptospires by real-time quantitative PCR. Journal of Medical Microbiology.

[bib30] Lin YP, Lee DW, McDonough SP, Nicholson LK, Sharma Y, Chang YF (2009a). Repeated domains of leptospira immunoglobulin-like proteins interact with elastin and tropoelastin. Journal of Biological Chemistry.

[bib31] Lin YP, Greenwood A, Nicholson LK, Sharma Y, McDonough SP, Chang YF (2009b). Fibronectin binds to and induces conformational change in a disordered region of leptospiral immunoglobulin-like protein B. Journal of Biological Chemistry.

[bib32] Lin YP, McDonough SP, Sharma Y, Chang YF (2011). Leptospira immunoglobulin-like protein B (LigB) binding to the C-terminal fibrinogen αC domain inhibits fibrin clot formation, platelet adhesion and aggregation. Molecular Microbiology.

[bib33] Malito E, Biancucci M, Faleri A, Ferlenghi I, Scarselli M, Maruggi G, Lo Surdo P, Veggi D, Liguori A, Santini L, Bertoldi I, Petracca R, Marchi S, Romagnoli G, Cartocci E, Vercellino I, Savino S, Spraggon G, Norais N, Pizza M, Rappuoli R, Masignani V, Bottomley MJ (2014). Structure of the meningococcal vaccine antigen NadA and epitope mapping of a bactericidal antibody. PNAS.

[bib34] Manford A, Xia T, Saxena AK, Stefan C, Hu F, Emr SD, Mao Y (2010). Crystal structure of the yeast Sac1: implications for its phosphoinositide phosphatase function. The EMBO Journal.

[bib35] Matsunaga J, Barocchi MA, Croda J, Young TA, Sanchez Y, Siqueira I, Bolin CA, Reis MG, Riley LW, Haake DA, Ko AI (2003). Pathogenic Leptospira species express surface-exposed proteins belonging to the bacterial immunoglobulin superfamily. Molecular Microbiology.

[bib36] McBride AJ, Cerqueira GM, Suchard MA, Moreira AN, Zuerner RL, Reis MG, Haake DA, Ko AI, Dellagostin OA (2009). Genetic diversity of the Leptospiral immunoglobulin-like (Lig) genes in pathogenic Leptospira spp. Infection, Genetics and Evolution.

[bib37] McWilliam H, Li W, Uludag M, Squizzato S, Park YM, Buso N, Cowley AP, Lopez R (2013). Analysis Tool Web Services from the EMBL-EBI. Nucleic Acids Research.

[bib38] Micsonai A, Wien F, Kernya L, Lee YH, Goto Y, Réfrégiers M, Kardos J (2015). Accurate secondary structure prediction and fold recognition for circular dichroism spectroscopy. PNAS.

[bib39] Morgan HP, Mertens HD, Guariento M, Schmidt CQ, Soares DC, Svergun DI, Herbert AP, Barlow PN, Hannan JP (2012). Structural analysis of the C-terminal region (modules 18-20) of complement regulator factor H (FH). PLoS One.

[bib40] Murray GL, King AM, Srikram A, Sermswan RW, Adler B (2010). Use of luminescent Leptospira interrogans for enumeration in biological assays. Journal of Clinical Microbiology.

[bib41] Mylonas E, Svergun DI (2007). Accuracy of molecular mass determination of proteins in solution by small-angle X-ray scattering. Journal of Applied Crystallography.

[bib42] Nielsen SS, Møller M, Gillilan RE (2012). High-throughput biological small-angle X-ray scattering with a robotically loaded capillary cell. Journal of Applied Crystallography.

[bib43] Palaniappan RU, McDonough SP, Divers TJ, Chen CS, Pan MJ, Matsumoto M, Chang YF (2006). Immunoprotection of recombinant leptospiral immunoglobulin-like protein A against Leptospira interrogans serovar Pomona infection. Infection and Immunity.

[bib44] Park KT, Burnett S, Davis WC (2015). Development and characterization of a monoclonal antibody specific for bovine CD209. Veterinary Immunology and Immunopathology.

[bib45] Petoukhov MV, Franke D, Shkumatov AV, Tria G, Kikhney AG, Gajda M, Gorba C, Mertens HD, Konarev PV, Svergun DI (2012). New developments in the ATSAS program package for small-angle scattering data analysis. Journal of Applied Crystallography.

[bib46] Petoukhov MV, Svergun DI (2015). Ambiguity assessment of small-angle scattering curves from monodisperse systems. Acta Crystallographica Section D Biological Crystallography.

[bib47] Picardeau M (2017). Virulence of the zoonotic agent of leptospirosis: still terra incognita?. Nature Reviews Microbiology.

[bib48] Ptak CP, Hsieh CL, Lin YP, Maltsev AS, Raman R, Sharma Y, Oswald RE, Chang YF (2014). NMR solution structure of the terminal immunoglobulin-like domain from the leptospira host-interacting outer membrane protein, LigB. Biochemistry.

[bib49] Putnam CD, Hammel M, Hura GL, Tainer JA (2007). X-ray solution scattering (SAXS) combined with crystallography and computation: defining accurate macromolecular structures, conformations and assemblies in solution. Quarterly Reviews of Biophysics.

[bib50] Rappuoli R, Bottomley MJ, D'Oro U, Finco O, De Gregorio E (2016). Reverse vaccinology 2.0: Human immunology instructs vaccine antigen design. The Journal of Experimental Medicine.

[bib51] Scarselli M, Aricò B, Brunelli B, Savino S, Di Marcello F, Palumbo E, Veggi D, Ciucchi L, Cartocci E, Bottomley MJ, Malito E, Lo Surdo P, Comanducci M, Giuliani MM, Cantini F, Dragonetti S, Colaprico A, Doro F, Giannetti P, Pallaoro M, Brogioni B, Tontini M, Hilleringmann M, Nardi-Dei V, Banci L, Pizza M, Rappuoli R (2011). Rational design of a meningococcal antigen inducing broad protective immunity. Science Translational Medicine.

[bib52] Silva EF, Medeiros MA, McBride AJ, Matsunaga J, Esteves GS, Ramos JG, Santos CS, Croda J, Homma A, Dellagostin OA, Haake DA, Reis MG, Ko AI (2007). The terminal portion of leptospiral immunoglobulin-like protein LigA confers protective immunity against lethal infection in the hamster model of leptospirosis. Vaccine.

[bib53] Skou S, Gillilan RE, Ando N (2014). Synchrotron-based small-angle X-ray scattering of proteins in solution. Nature Protocols.

[bib54] Vieira ML, Fernandes LG, Domingos RF, Oliveira R, Siqueira GH, Souza NM, Teixeira AR, Atzingen MV, Nascimento AL (2014). Leptospiral extracellular matrix adhesins as mediators of pathogen-host interactions. FEMS Microbiology Letters.

[bib55] Volkov VV, Svergun DI (2003). Uniqueness of *ab initio* shape determination in small-angle scattering. Journal of Applied Crystallography.

[bib56] Yan W, Faisal SM, McDonough SP, Divers TJ, Barr SC, Chang CF, Pan MJ, Chang YF (2009). Immunogenicity and protective efficacy of recombinant Leptospira immunoglobulin-like protein B (rLigB) in a hamster challenge model. Microbes and Infection.

