# Supplementary material for: Extended low-resolution structure of a Leptospira antigen offers high bactericidal antibody accessibility amenable to vaccine design
Source: eLife. 2017 Dec 6;6:e30051. doi: 10.7554/eLife.30051 (PMC5749957; doi:10.7554/eLife.30051)
Supplement: Figure 1—source data 1. [file elife-30051-fig1-data1.docx]

**Figure 1—source data 1**

| **LigB 5-domain Construct** | Conc. (mg/ml), [Dilution] | I(0), Guinier | I(0) / Dilution | Rg (Å), Guinier | Porod Volume (Å^3^) | MW (kDa), Porod Vol. | MW (kDa), Expected | Difference in MW (%) |
| --- | --- | --- | --- | --- | --- | --- | --- | --- |
| **LigB1-5** | 20.0 [3/3] | 9.090 | 9.090 | 51.47 | 53,141 | 45.1 | 46.2 | -2.4 |
| **LigB1-5** | [2/3] |  |  |  |  |  | 46.2 |  |
| **LigB1-5** | [1/3] |  |  |  |  |  | 46.2 |  |
| **LigB2-6** | 15.0 [3/3] | 5.228 | 5.228 | 58.43 | 54,453 | 46.3 | 46.4 | -0.2 |
| **LigB2-6** | 10.0 [2/3] | 3.412 | 5.117 | 56.09 | 52,340 | 44.6 | 46.4 | -3.9 |
| **LigB2-6** | 5.0 [1/3] | 1.628 | 4.884 | 55.98 | 52,026 | 44.0 | 46.4 | -5.2 |
| **LigB3-7** | 16.5 [3/3] | 6.162 | 6.162 | 57.01 | 60,683 | 51.2 | 46.6 | 9.9 |
| **LigB3-7** | 11.0 [2/3] | 3.398 | 5.097 | 58.57 | 63,648 | 54.2 | 46.6 | 16.3 |
| **LigB3-7** | 5.5 [1/3] | 1.642 | 4.925 | 57.72 | 63,812 | 57.1 | 46.6 | 22.5 |
| **LigB4-8** | 20.0 [3/3] | 2.153 | 2.153 | 57.57 | 55,751 | 47.5 | 46.5 | 2.2 |
| **LigB4-8** | [2/3] |  |  |  |  |  | 46.5 |  |
| **LigB4-8** | [1/3] |  |  |  |  |  | 46.5 |  |
| **LgB5-9** | 15.5 [3/3] | 4.889 | 4.889 | 69.26 | 77,628 | 65.5 | 47.1 | 39.1 |
| **LgB5-9** | 10.3 [2/3] | 2.867 | 4.300 | 66.07 | 75,590 | 63.6 | 47.1 | 35.0 |
| **LgB5-9** | 5.2 [1/3] | 1.273 | 3.820 | 63.37 | 71,566 | 61.2 | 47.1 | 29.9 |
| **LigB6-10** | 20.0 [3/3] | 2.981 | 2.981 | 52.55 | 54,598 | 46.4 | 47.4 | -2.1 |
| **LigB6-10** | 13.3 [2/3] | 1.855 | 2.782 | 50.95 | 52,799 | 43.9 | 47.4 | -7.4 |
| **LigB6-10** | 6.7 [1/3] | 0.811 | 2.432 | 53.80 | 54,313 | 46.4 | 47.4 | -2.1 |
| **LigB7-11** | 18.0 [3/3] | 5.360 | 5.360 | 51.84 | 58,088 | 48.9 | 47.5 | 2.9 |
| **LigB7-11** | 12.0 [2/3] | 2.864 | 4.296 | 53.29 | 58,574 | 49.4 | 47.5 | 4.0 |
| **LigB7-11** | 6.0 [1/3] | 1.292 | 3.876 | 51.98 | 55,626 | 46.5 | 47.5 | -2.1 |
| **LigB8-12** | 12.0 [3/3] | 9.460 | 9.460 | 47.77 | 55,267 | 46.1 | 47.3 | -2.5 |
| **LigB8-12** | [2/3] |  |  |  |  |  | 47.3 |  |
| **LigB8-12** | [1/3] |  |  |  |  |  | 47.3 |  |

**Table of LigB five domain construct SAXS profile values.**

The Guinier fits and Porod analysis were used to extract values related to the general shape of the proteins. The values in the table correspond to the concentration and corresponding dilution for each protein set, the Guinier-derived parameter for the intensity at q^2^ = 0 (I(0)), the intensity corrected by protein concentration (I(0) / dilution), the Guinier-derived parameter for the radius of gyration (Rg), the Porod Volume, the molecular weight (MW) based on the Porod Volume, the molecular weight (MW) expected for the primary sequence of the protein construct, and the percent difference that the Porod Volume MW is from the expected MW. Small differences (< ±10%) are shown in green to indicate the agreement between the experimental and predicted values. Larger differences (> ±10%) are shown in red and are suggestive of aggregation especially when values increase with increasing protein concentrations (e.g., LigB5-9). For LigB1-5, LigB4-8, and LigB12, only a single concentration was analyzed, but the molecular weights derived from these SAXS curves was consistent with predicted molecular weights. The accuracies of protein concentrations were not available for these experiments.
