# Supplementary material for: Extended low-resolution structure of a Leptospira antigen offers high bactericidal antibody accessibility amenable to vaccine design
Source: eLife. 2017 Dec 6;6:e30051. doi: 10.7554/eLife.30051 (PMC5749957; doi:10.7554/eLife.30051)
Supplement: Transparent reporting form [file elife-30051-transrepform.docx]

***eLife’s* transparent reporting form**

We encourage authors to provide detailed information *within their submission* to facilitate the interpretation and replication of experiments. Authors can upload supporting documentation to indicate the use of appropriate reporting guidelines for health-related research (see [EQUATOR Network](http://www.equator-network.org/%20)), life science research (see the [BioSharing Information Resource](https://biosharing.org/)), or the [ARRIVE guidelines](http://www.plosbiology.org/article/info:doi/10.1371/journal.pbio.1000412) for reporting work involving animal research. Where applicable, authors should refer to any relevant reporting standards documents in this form.

If you have any questions, please consult our Journal Policies and/or contact us: [editorial@elifesciences.org](mailto:editorial@elifesciences.org).

**Sample-size estimation**

- You should state whether an appropriate sample size was computed when the study was being designed
- You should state the statistical method of sample size computation and any required assumptions
- If no explicit power analysis was used, you should describe how you decided what sample (replicate) size (number) to use

Standard sample size or samples sizes that show statistically significant differences between experimental conditions and controls were used. Vaccine trials used a standard group size of six hamsters.

Please outline where this information can be found within the submission (e.g., sections or figure legends), or explain why this information doesn’t apply to your submission:

**Replicates**

- You should report how often each experiment was performed
- You should include a definition of biological versus technical replication
- The data obtained should be provided and sufficient information should be provided to indicate the number of independent biological and/or technical replicates
- If you encountered any outliers, you should describe how these were handled
- Criteria for exclusion/inclusion of data should be clearly stated
- High-throughput sequence data should be uploaded before submission, with a private link for reviewers provided (these are available from both GEO and ArrayExpress)

Please outline where this information can be found within the submission (e.g., sections or figure legends), or explain why this information doesn’t apply to your submission:

For SAXS scattering curves (Fig.1A, Fig.1-suppl.2A):

Scattering curves were collected on individual samples for 15 scans. Curves from matching buffer samples were also collected for 15 scans and subtracted from the sample to generate the final buffer-corrected scattering curve using the RAW software. This information is reported in the figure legends and within the material and methods.

For normalized P(*r*) plots (Fig.1B, Fig.1-suppl.1B):

The GNOM program from the ATSAS suite was used to determine P(*r*) plots. Optimal Rmax was determined by screening at 5 Å intervals and the P(*r*) plots were normalized to the first peak. This information is reported in the figure legends and within the material and methods.

For SAXS-derived envelopes (Fig.1C, Fig.1-suppl.2C):

SAXS profiles best representing the dilute (ideal) solution limit were used to generate 10 *ab initio* models and final averaged models with DAMMAVER and DAMMIF programs from the ATSAS suite. Models with a normalized spatial distribution > mean + 2*standard deviation were treated as outliers and not included in determining the final model. No more than one model was excluded for each structure. The averaged model for LigB5-9 was treated as an outlier based on the large % difference in MW vs. expected MW based on construct size and was omitted from the overlay in Fig. 1C. This information is reported in the figure legends and within the material and methods.

For NSD (Fig.1-suppl.1C):

The normalized spatial discrepancy was averaged from all 10 *ab initio* models using the ATSAS suite. This information is reported within the figure legend.

For Guinier plots (Fig.1-suppl.2B):

Guinier plots and fits were calculated with the RAW software using standard cutoffs. This information is reported within the figure legend.

For angles (Fig.1-suppl.2D):

The angles were measured from the dot representation of all available constructs. Three angles were measured for all points except for 1-2-3 and 10-11-12 where only one angle was included and for 2-3-4 and 9-10-11 where only two angles were included. This information is reported within the figure legend.

For ELISA binding screen for mAb to LigB1-7 or LigB7-12 (Fig.2B):

Three individual trials (biological replicates) of two replicates (technical replicates) were measured. For each trial, a mean value of the two replicates was obtained. The mean values for the three trials were used to obtain the overall mean values and statistics. This information is reported within the figure legend and the material and methods. A cutoff of OD_630_ = 1.0 was used to identify mAbs with moderate to high antigen binding strength.

For ELISA binding characterization of mAb to LigB1-7 and LigB7-12 (Fig.3), of mAb to LigB single and double domains and LigB chimeras (Fig.4A, Fig.4-suppl.1, Fig.6D-E) and of mAbs in sera to LigB single domains and LigB chimeras (Fig.7-suppl.2, Fig.7-suppl.3):

Three individual trials (biological replicates) were measured. Values from the three trials were used to obtain mean values and statistics. This information is reported within the figure legend and the material and methods. No values were treated as outliers.

For ELISA binding screen for mAb to LigB7-12 (Fig.5-suppl.3):

Two individual trials (biological replicates) of two replicates (technical replicates) were measured. For each trial, a mean value of the two replicates was obtained. The mean values for the two trials were used to obtain the overall mean values and statistics. This information is reported within the figure legend and the material and methods.

For flow cytometry (Fig.4B, Fig.4-suppl.2):

First, unstained *Leptospira* was identified by forward scatter (FSC) and side scatter (SSC). Second, selected mAb (C5) treated *L.* *interrogans* without secondary antibodies (FITC-conjugated goat anti-mouse IgG) was used as a negative control to set the gating region. For each sample, at least 20,000 cells were analyzed. Two independent trials (biological replicates) of two replicates (technical replicates) were measured. For each trial, a mean value of the two replicates was obtained. The mean values (MFI) for the two trials were used to obtain the overall mean values and statistics. This information is reported within the figure legend and the material and methods. No values were treated as outliers.

For dark-field microscopy (Fig.5A):

To count the motile *Leptospira* under the dark-field microscopy*,* each sample was measured separately by two researchers (blind). The mean value was obtained from the two independent (blind) measurements as a single technical replicate. Three independent trials (biological replicates) of two replicates (technical replicates) were measured. For each trial, a mean value of the two replicates was obtained. The mean values for the three trials were used to obtain the overall mean values and statistics. This information is reported within the figure legend and the material and methods. No values were treated as outliers.

For luminometer (Fig.5B):

Three independent trials (biological replicates) of two replicates (technical replicates) were measured. For each trial, a mean value of the two replicates was obtained. The mean values for the three trials were used to obtain the overall mean values and statistics. This information is reported within the figure legend and the material and methods. No values were treated as outliers.

For correlation plots (Fig.5D-E; Fig.5-suppl.4):

The mean values for ELISA antigen binding, for FACS cell binding, and for luminometer-derived LD_50_ are shown in correlation plots. Outliers were determined for LD_50_ plots using Cook’s Distance with a cutoff of 4/n.

For LT_50_ and LD_50_ (Fig.5-suppl.1):

The LT_50_ and LD_50_ values were obtained from fits to mean data point at each time and dose on plots of %survival vs. time and %survival vs. dose, respectively. The median lethal time (LT_50_) was calculated for individual mAbs from the fitted logistic curve using Origin software. The median lethal dose (LD_50_) was calculated for individual mAbs from the fitted dose inhibition curve using Origin software. The values for LT_50_ and LD_50_ are reported on the bar graph. This information is reported within the figure legend.

For average pairwise identity (Fig.6C):

The average percent identity for each LigB Ig-like domain is reported based on a pairwise matrix using alignments against each of the other 11 LigB Ig-like domains in LigB. This information is reported within the figure legend.

For hamster challenge trials (Fig.7A):

For each immunization group, six hamsters (biological replicates) were used to evaluate the protection efficacy. Survival rate was calculated by dividing the numbers of alive hamster by six at different time points. This information is reported within the figure legend.

For tissue-specific leptospiral load detection (Fig.7B):

Respective tissues from six hamsters in PBS group or LigB10-7-7 group were individually harvested. For each sample, qPCR analysis was performed in duplicate (technical replicates). The mean values from six hamsters (biological replicates) are reported and were also used to obtain the overall mean values and statistics. This information is reported within the figure legend and the material and methods. No values were treated as outliers.

For tissue slices and histopathological scoring (Fig.7C-H, Fig.7-suppl.4):

Respective tissues from four hamsters in each immunization group were individually harvested and fixed. Histopathological scoring was performed by board certificated veterinary pathologist, Dr. Sean McDonough. Representative tissue imagines from hamsters in the PBS group and the LigB10-7-7 group are presented. This information is reported within the figure legend.

For circular dichroism (Fig.7-suppl.1):

Three individual aliquots were measured for each sample. CD spectra curves are generated from mean values of the three individual measurements. This information is reported within the figure legend.

**Statistical reporting**

- Statistical analysis methods should be described and justified
- Raw data should be presented in figures whenever informative to do so (typically when N per group is less than 10)
- For each experiment, you should identify the statistical tests used, exact values of N, definitions of center, methods of multiple test correction, and dispersion and precision measures (e.g., mean, median, SD, SEM, confidence intervals; and, for the major substantive results, a measure of effect size (e.g., Pearson's r, Cohen's d)
- Report exact p-values wherever possible alongside the summary statistics and 95% confidence intervals. These should be reported for all key questions and not only when the p-value is less than 0.05.

Please outline where this information can be found within the submission (e.g., sections or figure legends), or explain why this information doesn’t apply to your submission:

(For large datasets, or papers with a very large number of statistical tests, you may upload a single table file with tests, Ns, etc., with reference to sections in the manuscript.)

For SAXS scattering curves (Fig.1A, Fig.1-suppl.2A):

Error bars depict standard deviation propagated according to standard procedures from the averaging of multiple scattering curves from the radially averaged images calculated by RAW and is noted within the figure legends and within the material and methods.

For NSD (Fig.1-suppl.1C):

Error bars depict the standard deviation for the NSD and is noted within the figure legend.

For ELISA binding screen for mAb to LigB1-7 or LigB7-12 (Fig.2B, Fig.5-suppl.3) and binding characterization of mAb to LigB1-7 and LigB7-12 (Fig.3), of mAb to LigB single and double domains and LigB chimeras (Fig.4A, Fig.4-suppl.1, Fig.6D-E) and of mAbs in sera to LigB single domains and LigB chimeras (Fig.7-suppl.2, Fig.7-suppl.3):

Error bars depict the standard deviation for the values from individual trials (biological replicates) and is noted within the figure legend. For Fig.4-suppl.1 and Fig.7-suppl.2, statistically significant binding levels were determined using a *t*-test. The *p*-value cutoffs are indicated for pair-wised comparisons with corresponding binding level of each sample with PBS only (Fig.4-suppl.1) and LigB12 (Fig.7-suppl.2).

For flow cytometry (Fig.4B, Fig.4-suppl.2):

Error bars depict the standard deviation for the values from individual trials (biological replicates) and is noted within the figure legend. Statistically significant binding levels were determined using a *t*-test. The *p*-value cutoffs are indicated for pair-wise comparisons of the MFI of bacteria with bound mAbs with corresponding MFI of bacteria with weakly bound mAbs or with only PBS.

For dark-field microscopy (Fig.5A) and luminometer (Fig.5B):

Error bars depict the standard deviation for the values from individual trials (biological replicates) and is noted within the figure legend. Statistically significant binding levels were determined using a *t*-test. The *p*-value cutoffs are indicated for pair-wise comparisons of mAb-treated group survival with corresponding survival of the pAb-treated group or the control groups.

For correlation plots (Fig.5D-E; Fig.5-suppl.4):

The R^2^ value for linear fits is reported in the figure legend for data sets including (Fig.5D-E) and excluding outliers (all).

For average pairwise identity (Fig.6C):

Error bars depict the standard deviation for average percent identity is noted within the figure legend.

For tissue-specific leptospiral load detection (Fig.7B):

The large bar depicts the mean values for six hamsters (biological replicates) while the error bars depict the standard deviation of these mean values. This information is noted within the figure legend.

**Group allocation**

- Indicate how samples were allocated into experimental groups (in the case of clinical studies, please specify allocation to treatment method); if randomization was used, please also state if restricted randomization was applied
- Indicate if masking was used during group allocation, data collection and/or data analysis

No special group allocation was used.

Please outline where this information can be found within the submission (e.g., sections or figure legends), or explain why this information doesn’t apply to your submission:

**Additional data files (“source data”)**

- We encourage you to upload relevant additional data files, such as numerical data that are represented as a graph in a figure, or as a summary table
- Where provided, these should be in the most useful format, and they can be uploaded as “Source data” files linked to a main figure or table
- Include model definition files including the full list of parameters used
- Include code used for data analysis (e.g., R, MatLab)
- Avoid stating that data files are “available upon request”

No additional data source files have been deposited. The following previously reported data was utilized in the production of this manuscript.

Figure 1 is connected to Figure 1-source data 1.

The source for the sequence of *Leptospira interrogans* serovar Pomona (GenBank^TM^, FJ030916) is noted in the material and methods. Reference: Chang, Y.-F. (2008) FJ030916: *Leptospira interrogans* serovar Pomona isolate pLPLIGB LigB (ligB) gene, partial cds. GenBank (http://www.ncbi.nlm.nih.gov/nuccore/FJ030916).

The source for the NMR structure of LigB12 (PDB ID 2MOG) is noted in figure legends for Fig.4B and Fig.4-suppl.1 and also the material and methods. Reference: Ptak, C. P.; Hsieh, C.; Lin, Y.; Maltsev, A. S.; Raman, R.; Sharma, Y.; Oswald, R. E.; Chang, Y. (2014) 2MOG: Solution structure of the terminal Ig-like domain from *Leptospira interrogans* LigB. Worldwide Protein Data Bank (http://www.rcsb.org). DOI: 10.2210/pdb2mog/pdb

Please indicate the figures or tables for which source data files have been provided:
